# Convolutional automatic identification of B-lines and interstitial syndrome in lung ultrasound images using pre-trained neural networks with feature fusion

**DOI:** 10.3389/fdgth.2025.1632376

**Published:** 2026-01-19

**Authors:** Khalid Moafa, Maria Antico, Damjan Vukovic, Christopher Edwards, David Canty, Ximena Cid Serra, Alistair Royse, Colin Royse, Kavi Haji, Jason Dowling, Marian Steffens, Davide Fontanarosa

**Affiliations:** 1 School of Clinical Sciences, Queensland University of Technology, Brisbane, QLD, Australia; 2 College of Applied Medical Sciences, Jazan University, Jazan, Saudi Arabia; 3 Centre for Biomedical Technologies (CBT), Queensland University of Technology, Brisbane, QLD, Australia; 4 Australian e-Health Research Centre, The Commonwealth Scientific and Industrial Research Organisation (CSIRO), Brisbane, QLD, Australia; 5 Department of Surgery (Royal Melbourne Hospital), University of Melbourne, Parkville, VIC, Australia; 6 Department of Cardiothoracic Surgery, Royal Melbourne Hospital, Melbourne, VIC, Australia; 7 Department of Anaesthesia and Pain Management, Royal Melbourne Hospital, Melbourne, VIC, Australia; 8 Department of Intensive Care, Peninsula Health, Frankston, VIC, Australia

**Keywords:** interstitial syndrome, lung ultrasound, deep learning, transfer learning, features fusion

## Abstract

**Introduction:**

Interstitial/alveolar syndrome (IS) is a condition detectable on lung ultrasound (LUS) that indicates underlying pulmonary or cardiac diseases associated with significant morbidity and increased mortality rates. However, diagnosing IS using LUS can be challenging and time-consuming, and it requires clinical expertise.

**Methods:**

In this study, multiple convolutional neural network (CNN) models were trained as binary classifiers to accurately screen for IS in LUS frames by distinguishing between IS-present and healthy cases. The CNN models were initially pre-trained using a generic image dataset to learn general visual features (ImageNet) and then fine-tuned on our specific dataset of 108 LUS clips from 54 patients (27 healthy and 27 with IS, two clips per patient) to perform a binary classification task. Each clip in the dataset was assessed by a clinical sonographer to determine the presence of IS features or confirm healthy lung status. The dataset was split into training (70%), validation (15%), and testing (15%) sets.

**Results:**

Following the process of fine-tuning, we successfully extracted features from pre-trained DL models. These extracted features were then utilised to train multiple machine learning (ML) classifiers, resulting in significantly improved accuracy in IS classification compared with the individual CNN models. Advanced visual interpretation techniques such as heatmaps based on gradient-weighted class activation mapping (Grad-CAM) and local interpretable model-agnostic explanations (LIME) were implemented to further analyse the outcomes. The best-trained ML model achieved a test accuracy rate of 98.2%, with specificity, recall, precision, and F1 score values above 97.9%.

**Conclusion:**

Our study demonstrates the feasibility of using a pre-trained CNN as a diagnostic tool for IS screening on LUS frames, integrating targeted data filtering, feature extraction, and fusion techniques. The data-filtering technique refines the training dataset by excluding LUS frames that lack IS-related features (e.g., absence of B-lines). Feature fusion combines features learnt from different models or “fused” to enhance overall predictive performance. This study confirms the practicality of using pre-trained CNN models with feature extraction and fusion techniques for screening IS using LUS frames. This represents a noteworthy advancement in improving the efficiency of diagnosis. In the next steps, validation on larger datasets will assess the applicability and robustness of these CNN models in more complex clinical settings.

## 1 Introduction

Lung ultrasound (LUS) has gained clinical acceptance for diagnosing and managing lung diseases because of its advantages over conventional tests such as computed tomography (CT) and benefits such as accessibility, absence of radiation risk, and portability (1). These benefits make it ideal for emergency and intensive care settings (2, 3). However, LUS is operator-dependent, and LUS training can be costly and time-consuming, often restricted to clinicians who have access to such training (4).

Deep learning (DL) algorithms have been developed to enable computer-automated diagnosis of pleural effusion and consolidation (4–6). Recent advances in DL and convolutional neural networks (CNNs) have been achieved by using the expertise of LUS-trained clinicians as a reference for DL algorithms in the analysis and recognition of LUS patterns (7, 8). This technological advancement assists in reducing risks of operator-related overlooks or misdiagnoses and potentially provides untrained clinicians with a diagnostic ultrasound (US) tool that is reasonably accurate.

High-resolution computed tomography (HRCT) remains the gold-standard diagnostic tool for interstitial/alveolar syndrome (IS) (9). However, limited access and exposure to risks related to transportation and exposure to ionizing radiation make HRCT less desirable in critical care. LUS has been demonstrated to be superior to chest X-ray in assessing lung pathologies such as pulmonary oedema, pleural effusion, pneumonia, and interstitial lung disease (ILD) (10). It is particularly valuable for expediting diagnosis and enabling timely treatment initiation (11).

The interpretation of LUS images largely relies on artefact analysis, which has been shown to correlate with CT findings (9). B-lines are reverberation artefacts in the form of vertical, laser-like, mobile lines that indicate interferences resulting from interstitial fluid, inflammation, or fibrosis (12). The diagnosis of IS is appropriate when 3 or more B-lines are present within a single intercostal space and in non-dependent parts of the lungs; however, the significance varies based on the clinical context of the presentation. Bilateral IS can be caused by cardiogenic pulmonary oedema, interstitial lung diseases such as pulmonary fibrosis, or viral pneumonitis, including COVID-19 (13). Conversely, localised IS may indicate an early stage of pneumonia. Evidence shows that identifying and quantifying B-lines not only aids in diagnosing cardiogenic pulmonary oedema but also guides treatment and its response through repeated scanning and may provide prognostic information (14).

This study demonstrates the implementation and training of DL models, specifically CNNs, to automate B-line detection on US images in patients with IS. Currently, DL approaches, particularly involving the use of CNNs, have been demonstrated to be effective for a wide range of pathologies in LUS (15, 16). CNNs are able to automatically and robustly learn specific characteristics of the images, allowing them to reliably detect (17), segment (5), and classify (4, 6) multiple LUS pathologies. It is well known that DL models require large amounts of labelled data for training (18, 19). Transfer learning (TL) is a possible approach proposed to deal with “data starvation” problems, as it can compensate for a lack of data in a target domain by inheriting or maintaining the knowledge learnt in a data-rich source domain (20). According to the literature, using pre-trained CNNs, such as ImageNet models, as feature extractors or fine-tuning pre-trained CNNs can improve performance for various medical image analysis tasks compared with a DL model that is built without pre-existing features (21, 22).

Addressing the pressing need for automated LUS analysis tools that accurately and timely detect IS, thereby significantly reducing diagnostic subjectivity, facilitating early disease identification, and potentially leading to improved patient outcomes, forms the core motivation for this work. The novelty of this work lies in applying DL pre-trained models, namely Xception and InceptionResnetV2, which were initially trained on the ImageNet dataset, to a unique IS dataset and training these models on different data-filtering techniques. In addition, we implemented a feature fusion technique to further improve the performance of the DL models by combining features derived from these models. The combined features were further utilised to train multiple classifiers to achieve high diagnostic accuracy (23). We also interpreted the complexity of the “black box” of the DL models used by utilising visualisation and interpretation techniques such as gradient-weighted class activation mapping (Grad-CAM) and local interpretable model-agnostic explanations (LIME) (24–26).

## 2 Methods

### 2.1 Dataset

**Figure 1:**
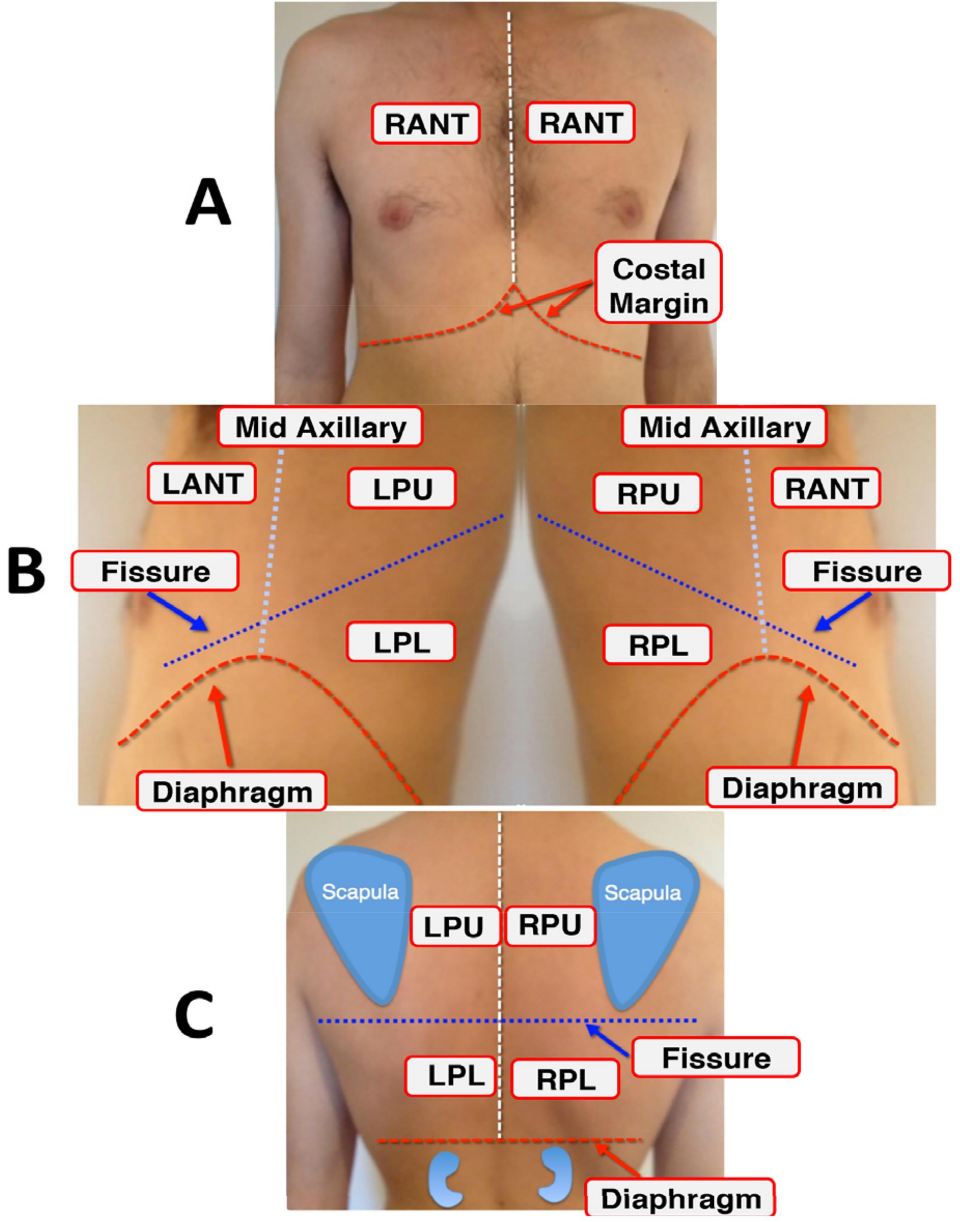
The six lung scanning zones: **(A)** displaying right anterior (RANT) and left anterior (LANT) views; **(B)** encompassing left posterior upper (LPU), left posterior lower (LPL), right posterior upper (RPU), and right posterior lower (RPL) views; **(C)** providing a posterior view for LPU, RPU, RPL, and LPL.

**Figure 2:**
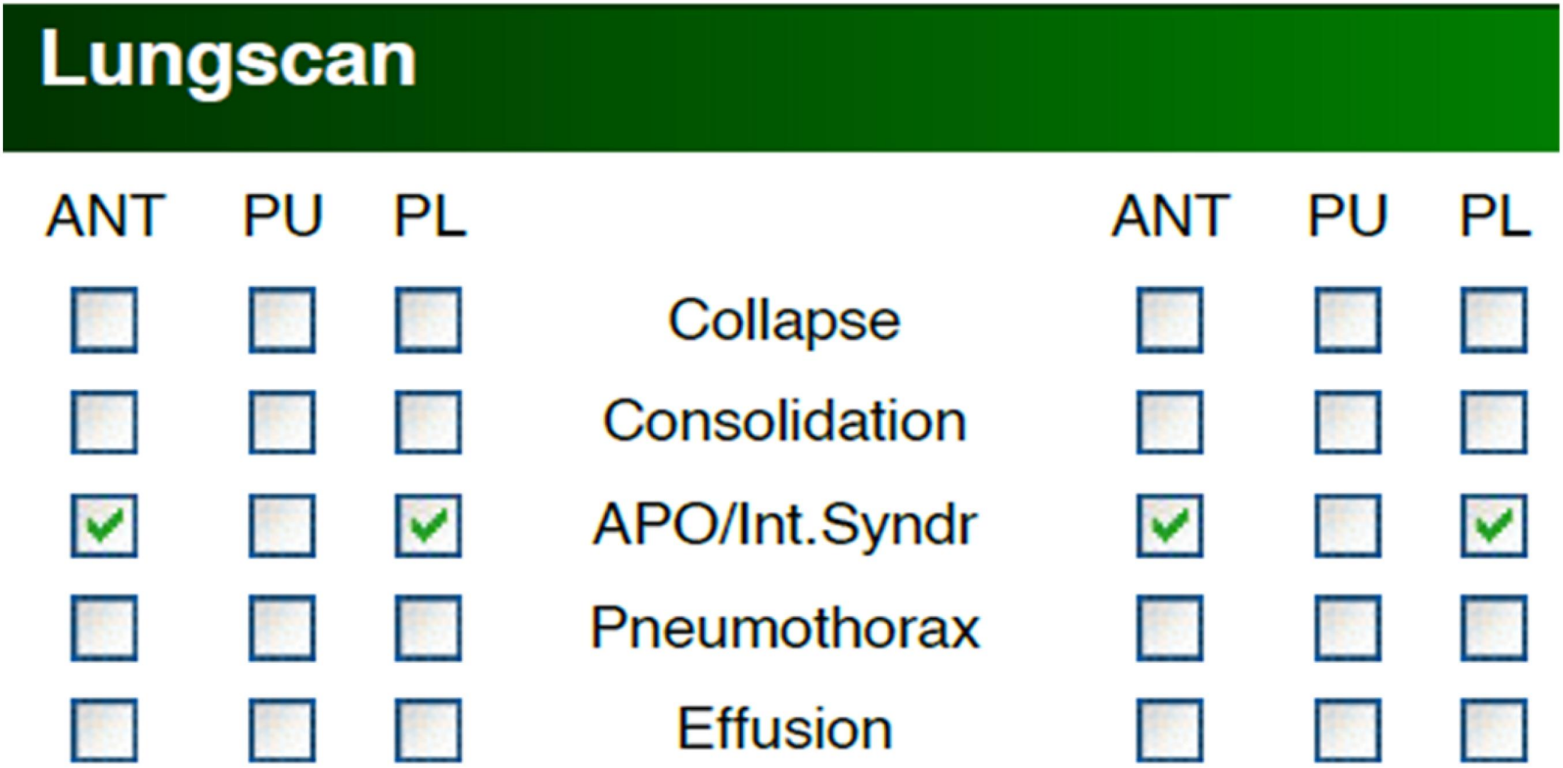
An example of data in the medical report, indicating six LUS scanning regions corresponding to each LUS pathology. In this example, regions are marked with IS and APO (acute pulmonary oedema). The right side of the image corresponds to the left side of the patient (LANT and LPL), while the left side of the image corresponds to the right side of the patient (RANT and RPL).

**Figure 3:**
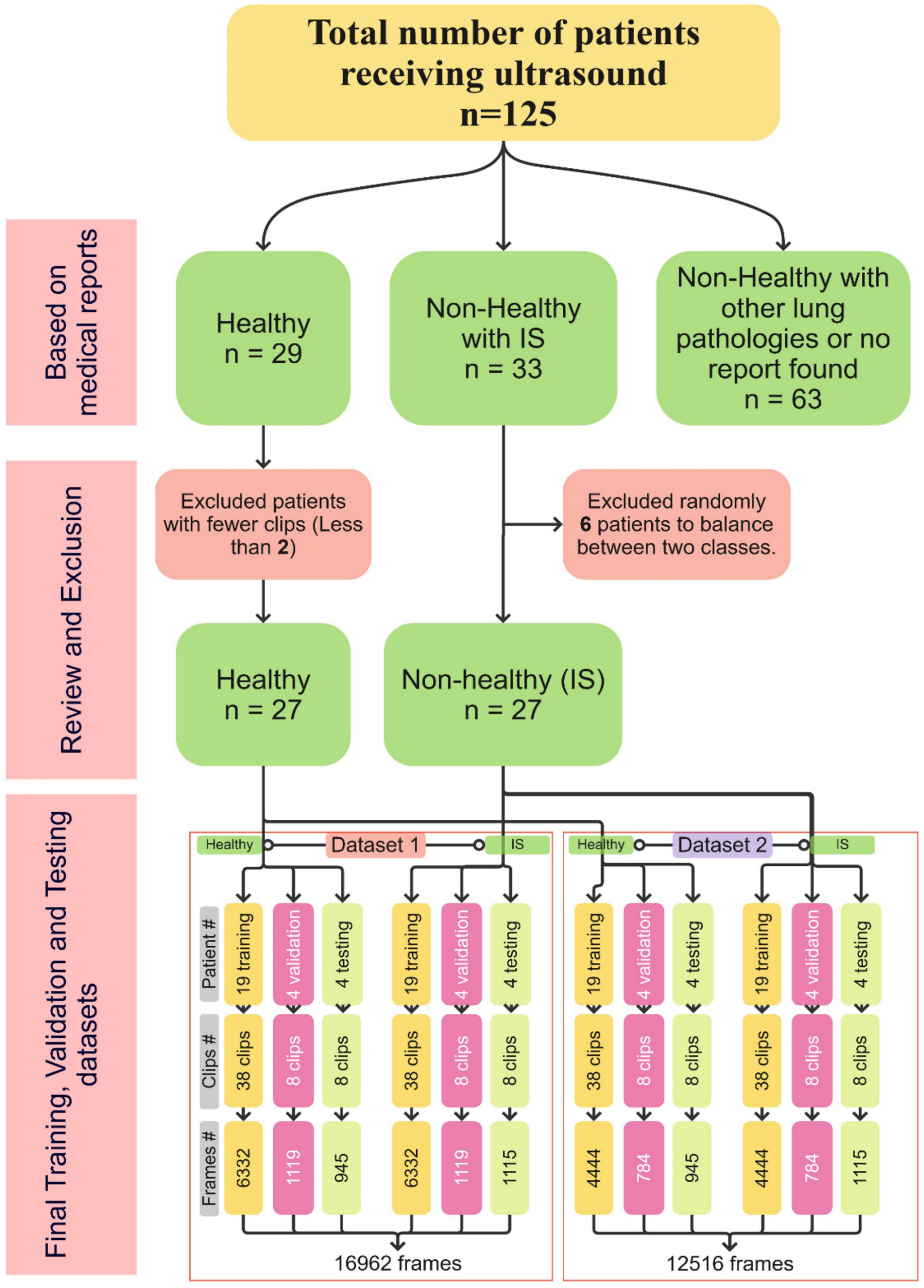
Distribution of patient numbers, number of clips, and number of frames, along with the process of reviewing, excluding, and finalising training, validation, and testing sets for both Dataset 1 and Dataset 2.

**Figure 4:**
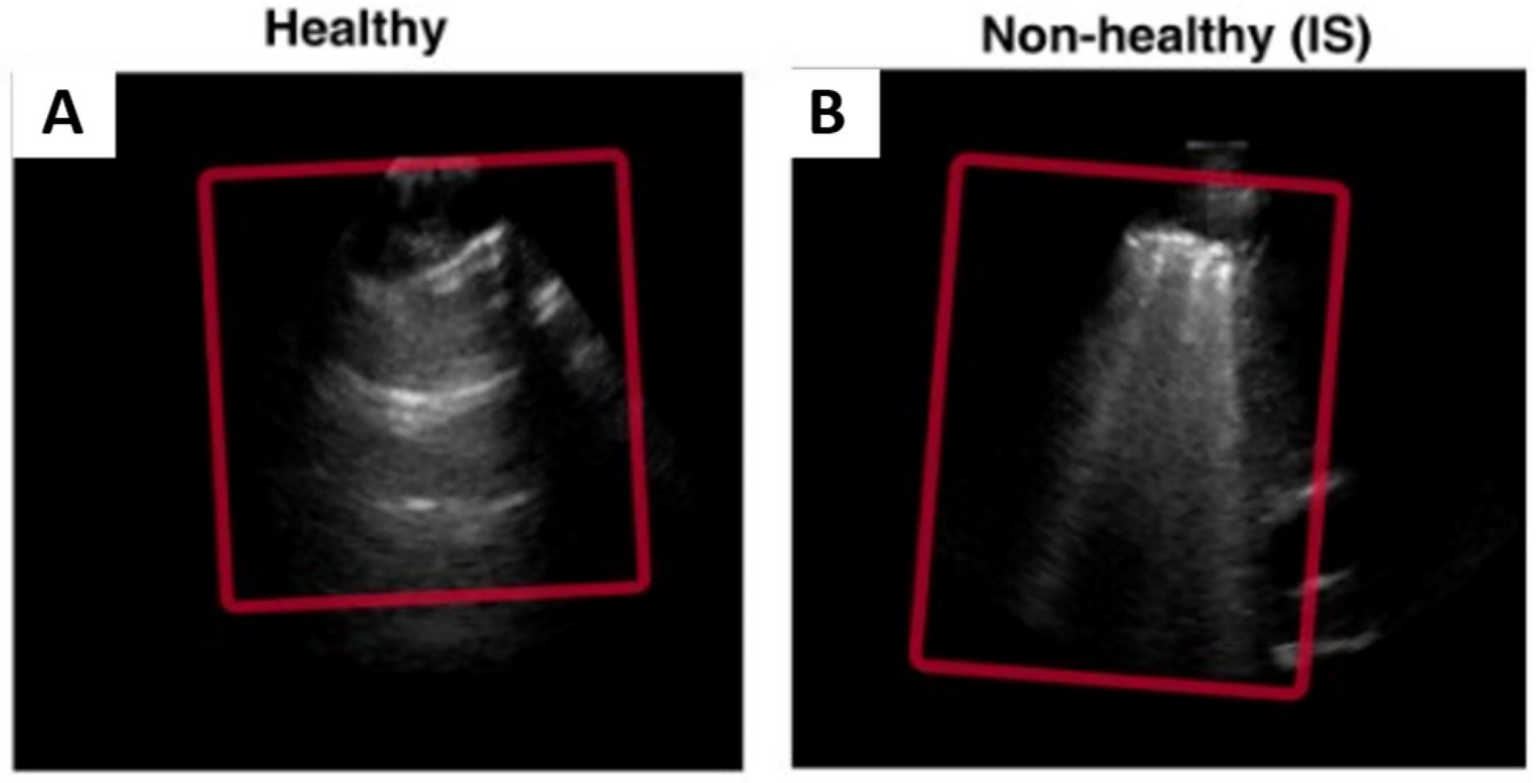
The displayed images are LUS examples of healthy **(A)** and IS **(B)** frames. On the left is a healthy frame showing A-lines (horizontal lines). On the right is a non-healthy frame with B-lines indicating non-healthy lungs (vertical lines).

The LUS datasets used were fully anonymised and were collected at the Royal Melbourne Hospital. The study was approved by the Melbourne Health Human Research Ethics Committee (HREC/18/MH/269) (27). The US dataset comprised 125 patients for a total of 1,034 LUS clips. At least six unique lung scanning zones were evaluated and labelled (Figure 1) by our clinical experts (DC and XC) following the protocol shown in Figure 2. The initial dataset included clips from 54 unidentified patients, 27 healthy, and 27 with IS labelled as “non-healthy.” The total number of LUS clips included were 108, comprising 16,962 LUS frames (8,481 frames each for healthy and IS patients) (Figure 3). Two LUS examples of IS and healthy frames are demonstrated in Figures 4A, B, respectively.

### 2.2 Filtering techniques

**Figure 5:**
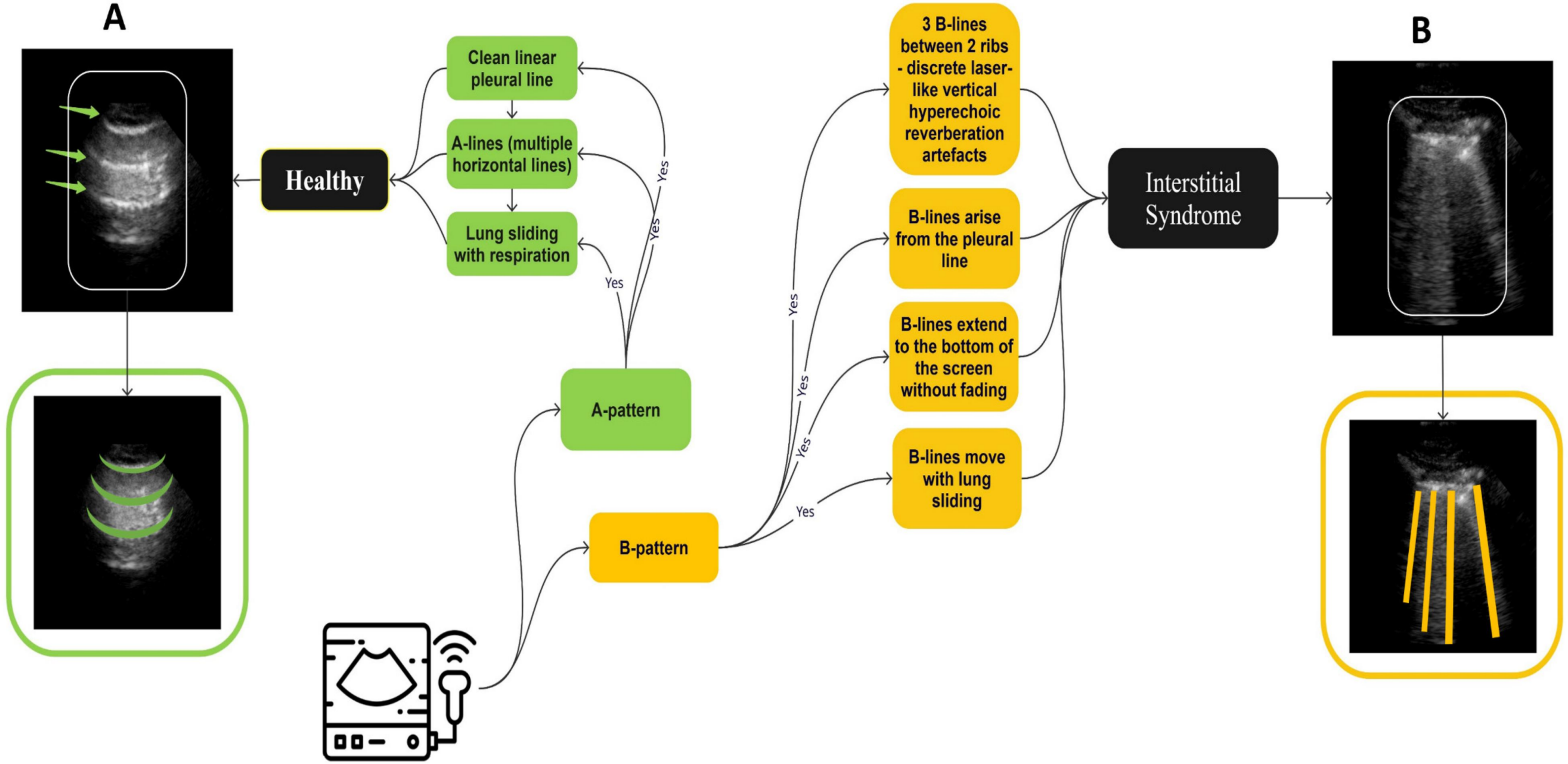
A summary of recommendations for diagnosing IS and healthy LUS clips. **(A)**, A healthy condition (green), is characterised by the presence of A-lines, with a clear linear pleural line and lung sliding during inspiration. Conversely, **(B)**, IS (yellow) is characterised by the presence of three B-lines between two ribs, which are associated with four main features: the B-lines exhibit a laser-like appearance, arise from the parietal line, reach the bottom of the screen without fading, and move with lung sliding and lung pulse.

The filtering techniques applied in this study are as follows: Scenario 1 involves the thorough inclusion of all LUS frames from all clips in the training datasets (Dataset 1). In contrast, Scenarios 2 and 3 utilise a selective filtering technique to refine the training dataset (Dataset 2) by excluding LUS frames that do not exhibit the main IS features (i.e., absence of B-lines), thereby prioritising clinically relevant features. The two datasets are illustrated in Figure 3. Based on predefined clinical criteria, all LUS clips were labelled as healthy or non-healthy (IS cases) (Figure 5). No other lung pathologies (e.g., pleural effusion, consolidation, or atelectasis) were included in the dataset. These criteria were adapted from international evidence-based recommendations for point-of-care ultrasound (POCUS) (28). It is crucial to note that, in the clip-based labelling method used in this study, a clip classified as IS may include individual frames that do not exhibit IS features and could be deemed healthy. This observation highlights the natural variation and complexity of LUS, emphasising that not all frames from IS clips will consistently show the exact features associated with IS.

### 2.3 Implementation of the models

The LUS dataset was divided into three subsets for effective model development and performance assessment: training (70% ≈ 76 LUS clips), validation (15% ≈ 16 LUS clips), and test sets (15% ≈ 16 LUS clips). Three different approaches were followed, referred to as Scenarios 1, 2, and 3, with distinct pre-processing techniques and pre-trained models. In Scenario 1, the Xception pre-trained model was employed (29). All LUS frames were included in the training and validation process, incorporating the total number of healthy and non-healthy frames from all clips (14,902 frames, referred to as Dataset 1). Scenario 2 entailed the utilisation of Xception and Inceptionresnetv2 pre-trained models. A more selective strategy was adopted in Scenario 2 (10,546 frames; referred to as Dataset 2), where non-healthy frames were re-evaluated, and any frames exhibiting characteristics not conducive to the main features characterising IS (i.e., absence of B-lines) were excluded. This selection criterion was applied to ensure that only relevant features were included in the training dataset. In Scenario 3, an Xception model without pre-existing features was trained (non-pre-trained model). It learnt features and weights exclusively from the LUS data without any TL from general image knowledge. Furthermore, similar to Scenario 2, data filtering was applied to the non-pre-trained model, with an evaluation of only non-healthy frames and the exclusion of any frames exhibiting characteristics not conducive to the main features characterising IS (absence of B-lines) within the training dataset. The pre-trained models, Xception and InceptionResNetV2, were selected for their proficiency in medical image classification gained through training on the ImageNet dataset with over 14 million natural images across more than 20,000 classes (29, 30).

To adapt the DL models to our IS detection task, with two classes, IS and healthy class, the architectures of all models (Xception, Inceptionresnetv2, and non-pre-trained model) were customised. The top layer of the models, known as the classifier, which was originally designed to classify 1,000 different classes (such as animals or household items), was replaced with a two-class classifier. The LUS images were also downsized from 720 × 920 pixels to 299 × 299 pixels to align with the input dimensions specified by the models' architectures. The resizing was performed using bilinear interpolation in MATLAB software (Version R2023b). The aspect ratio of the LUS images was preserved during resizing to avoid distortion of anatomical features.

**Figure 6:**
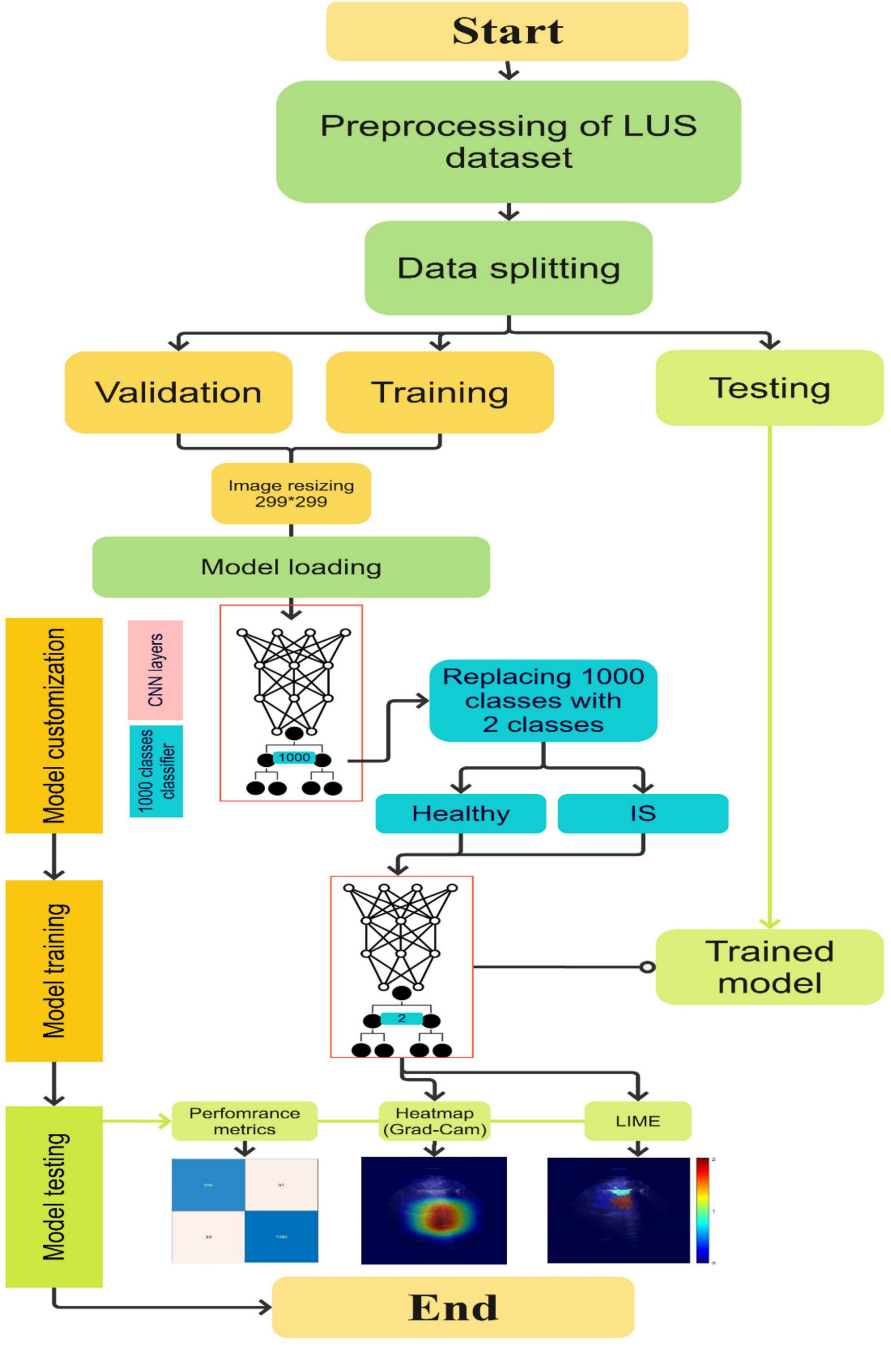
A flowchart demonstrating the process of model customisation, training, and evaluation used in the study.

The Xception model has about 170 layers and 22.9 million trainable parameters. It uses depth-wise separable convolutions across 14 modules to improve feature extraction. The InceptionResnetV2 model, with a more complex structure, includes 843 layers and 55.8 million trainable parameters, combining the Inception and ResNet architectures. As the non-pre-trained model, we employed a modified version of the Xception model that was devoid of its pre-trained weights. The MATLAB software was used to run the models and monitor the training and testing process. The models were trained on a graphics processing unit with an NVIDIA TITAN RTX and 25 GB RAM, running Ubuntu 20.04.6 LTS. The Adam optimiser was used during training. The configuration of training code and model customisation steps was guided by the available code from the GitHub repository by Alzubaidi et al. (31). After training, the models were tested on the test subset, and their performance was evaluated using multiple performance metrics. Figure 6 illustrates the workflow followed to train and test the models, which includes data processing, model customisation, and model performance evaluation tools. In addition, Table 1 outlines the model hyperparameters.

**Table 1:** The DL parameters used in the training and validation process of the models.

Parameter	Value
Batch size	10
Epochs	10
Shuffle	Every epoch
Learning rate	10^−4^
Optimizer	Adam
Image size	299*299

### 2.4 Explainability and interpretability of DL models

**Figure 7:**
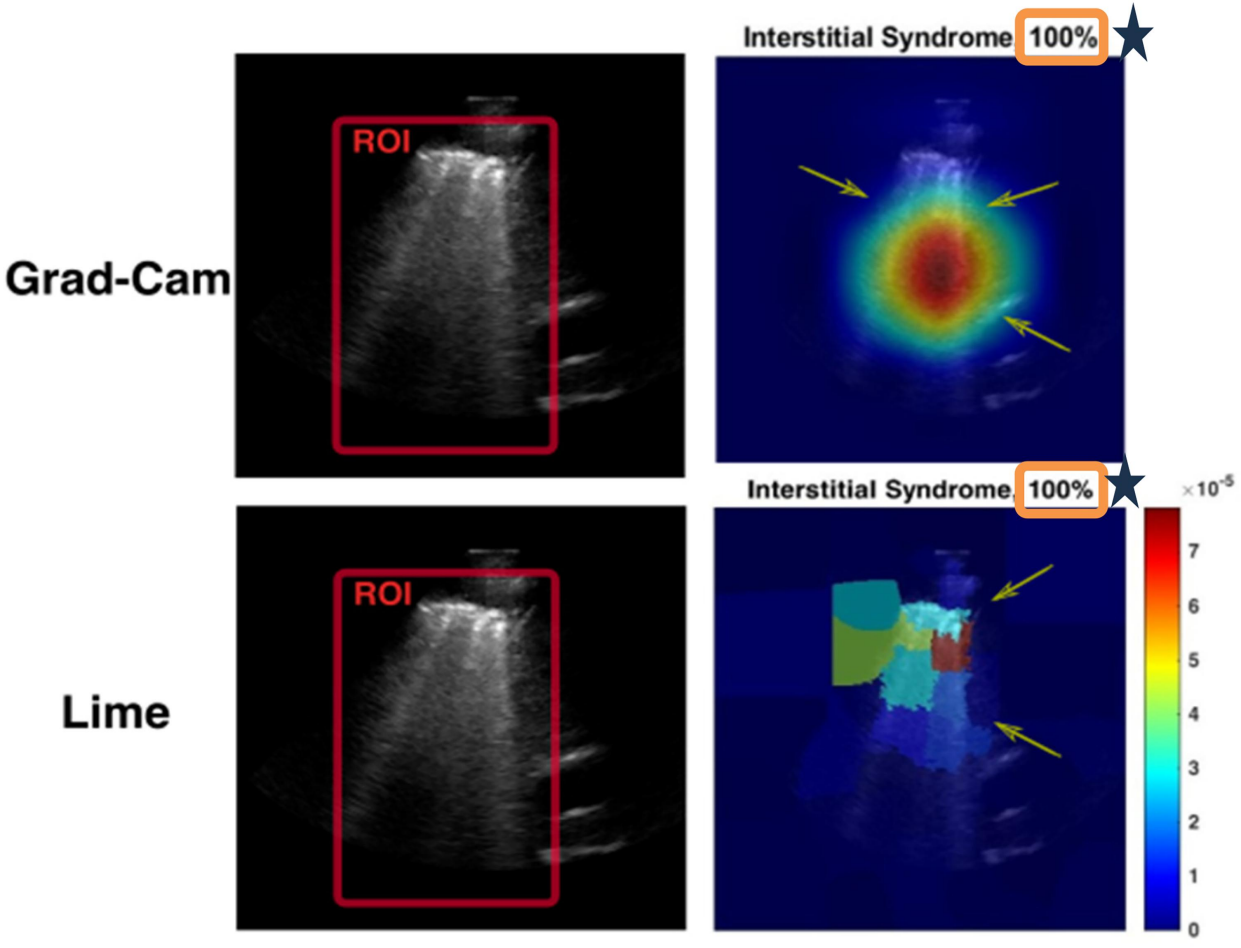
Examples of Grad-CAM and LIME plots. These plots show clearly how the model focuses on the ROI and what features and regions are important for the model's prediction. In the case of Grad-CAM, the heatmap pinpointing significantly impacted the prediction, whereas the red zones represent the elements the model focused on most. For LIME, features with higher scores, and thus more intense colours, are the ones that the model considered more important in making its decision. For example, the green and red colour areas (ranging from 3 to 10^−5^ on the heatmap scale on the LIME image) indicate the most important areas that support the decision made by the model. Conversely, the blue areas (0–3) outline the less important features of the model's decision. In addition, at the top of the visualisation plots, the prediction comes with a confidence score (100%), which indicates the model's certainty about its prediction.

The Grad-CAM visualisation technique was used in our model evaluation to enhance the explainability of model predictions (26). Grad-CAM provides a visual explanation in the form of a heatmap overlay on the image, highlighting the region of interest (ROI) in the output image (26), which refers to a specific area within an LUS image used to identify particular pathologies or diseases. For instance, in cases of non-healthy frames (IS) (Figure 7. Grad-CAM), the ROI may be defined as an area containing B-lines. This technique generates heatmaps, overlaying the original image to highlight areas influencing the model's decision and aiding in identifying related features. In addition, LIME was used to explain predictions by estimating the decision boundary in a specific input image, focusing on the intended ROI in the LUS image, and generating a heatmap scale (Figure 7, LIME).

LIME highlights influential regions that contribute to a specific prediction, aiding understanding of the model's decision-making process (24). It approximates the boundary that defines the ROI by creating a new scale for a heatmap. This scale highlights the regions of the input image that have the most impact on the model's prediction. LIME divides the image into identifiable portions and evaluates the impact of each part of the LUS image. In Figure 7, the LIME visualisations show the most important features, which are represented by scores and colours. Higher scores, indicated by more intense colours (from blue to red), correspond to features that contributed more significantly to the model's decision.

A smaller, representative subset of frames was randomly selected from the test set (2,060 LUS frames) for evaluation. Specifically, 20% of the total dataset (412 out of 2,060) was sampled by selecting one frame every five frames using a simple MATLAB script. Grad-CAM and LIME were generated to analyse which areas of the images the model focused on, producing corresponding cropped images for each frame. Each sampled frame was manually reviewed to determine whether the Grad-CAM or the LIME visualisations correctly highlighted the intended ROI. For IS frames, correct localisation was defined as “accurate emphasis on B-lines or pleural irregularities”; for healthy frames, correct localisation was defined as “emphasis on normal pleural and A-line features.” Grad-CAM and LIME accuracies were then computed as the proportion of frames correctly localised with respect to the expert-defined ROI.

Along with Grad-CAM and LIME plots, the confidence score was used. The confidence value, or the probability score, quantifies the model's level of confidence in its predictions (25). Higher probabilities or confidence values generally indicate higher confidence in the model (i.e., a confidence score of 100% indicates that the model has absolute certainty in its prediction). In comparison, lower probabilities suggest lower confidence in the model (25). A confidence score of 50% means that the model is equally likely to be correct or incorrect. All developed models were tested on the unseen test dataset to generate Grad-CAM and LIME plots, along with the corresponding confidence scores (Figure 7).

Nevertheless, a high level of confidence does not guarantee the accuracy of the prognosis. The model's indication solely reflects its confidence level derived from the knowledge acquired during training. The model's confidence may be significantly high. Yet, it can produce an inaccurate clinical diagnosis, particularly if it has been trained on biased data or encounters data that significantly deviate from its training set (32).

### 2.5 Feature fusion technique

The feature fusion process in artificial intelligence (AI) combines information from multiple AI models trained on the same dataset using different ML classifiers (33). This strategy is a powerful technique employed to enhance overall performance by incorporating features from different DL models. Its objective is to acquire and merge additional knowledge from multiple models in order to improve the representation of the features extracted from them (34). During the learning stage, the initial layers of each DL model acquire low-level features such as colours, edges, and forms, while the last layers acquire the high-level features of an object. Consequently, the model's final output features result from this hierarchical learning process, in which complex, high-level features are built upon more fundamental ones. Features are extracted from the bottleneck layers, which are the layers prior to the output layer. These layers are rich in complex features that have been analysed through the network and are considered highly informative for the classification task (35). Feature fusion is then utilised, where features from the bottleneck layers learnt from different models are combined or “fused.”

After the feature extraction phase, the extracted features undergo a process of normalisation to ensure that they are on a comparable scale, followed by concatenation to form a unified feature vector for each image. This combination offers an improved depiction of the features and enables a more thorough representation of the underlying patterns and features in the data. These fused features are then used as inputs to train a machine learning (ML) classifier, which adjusts its parameters by comparing predictions to ground truth (GT) labels to minimise prediction errors. This method enables ML classifiers to leverage the capabilities and distinctive attributes of each DL model, thereby improving understanding of the target tasks (36). The integration of features from different models provides numerous benefits for ML classifiers. In the present study, the built-in Classification Learner in MATLAB 2023b was utilised to develop ML classifiers, which include linear discriminant analysis, neural networks, coarse KNN, cubic SVM, the boosted tree, and the coarse tree, to determine the most efficient classifier for this detection task.

**Figure 8:**
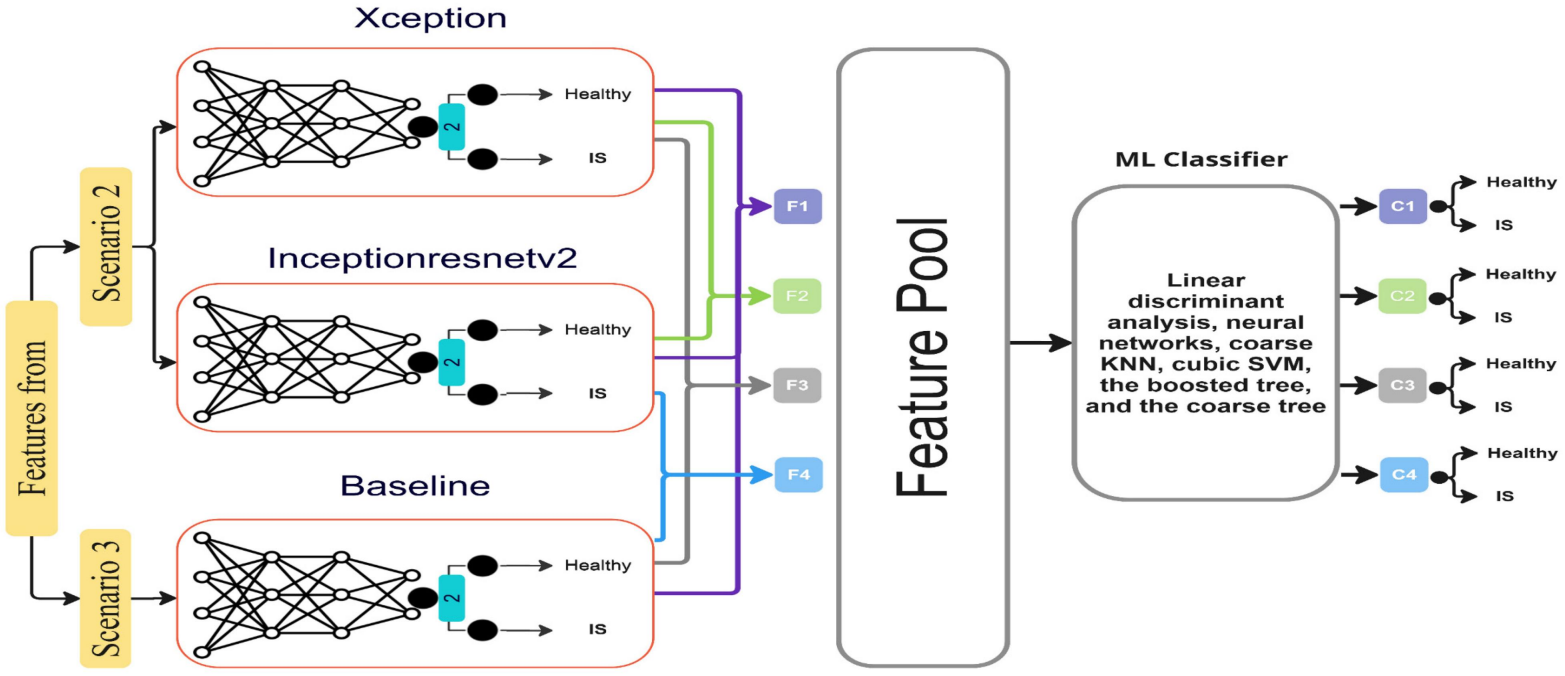
Four fusion processes (F1, F2, F3, and F4) that combine the features of different models from Scenario 2 and Scenario 3 are shown: F1 (feature fusion of Xception, InceptionResnetV2, and non-pre-trained Xception model), F2 (feature fusion of Xception and InceptionResnetV2), F3 (fusion of non-pre-trained Xception model and pre-trained Xception), and F4 (fusion of non-pre-trained Xception model and InceptionResnetV2). Features extracted from these models (F1, F2, F3, and F4) are pooled together and fed into various machine learning (ML) classifiers such as linear discriminant analysis, neural networks, KNN, cubic SVM, the boosted tree, and the coarse tree. These classifiers then make final predictions (C1, C2, C3, and C4).

The study utilised multiple models for this task, beginning with a comprehensive fusion (F1) involving all models mentioned in Scenarios 2 and 3, namely Xception, InceptionResnetV2, and the non-pre-trained model. A feature fusion (F2) was also performed with the two best models identified in Scenario 2. Furthermore, two separate feature fusion processes were performed: one between the non-pre-trained model and Xception (F3) and another between the non-pre-trained model and InceptionResnetV2 (F4), each done individually. Each feature fusion process generated fused features, which were then used as input to the ML classifiers for training and performance evaluation, as shown in Figure 8. The resulting classifiers are named C1, C2, C3, and C4.

### 2.6 Comparison of AI models with medical experts

To assess the robustness of our developed models for IS detection, a comparative analysis was conducted against clinical diagnoses made on individual clips. A randomly selected test set of 16 video clips representing our dataset was assessed. The GT labels were established during data acquisition by two senior clinicians (DC and XC) and ensured expert consensus. Each LUS clip was reviewed and labelled as healthy or interstitial syndrome (IS) according to predefined diagnostic criteria described earlier (Section 2.3, Figure 5). These LUS labels were documented and served as the GT for model training and evaluation. A blinded review was further conducted by two additional experts (MS and CE), each with 15 years of experience and holding senior positions at the Queensland University of Technology. They independently labelled the clips as 0 (healthy) or 1 (IS). Their diagnoses served as a reference point for evaluating the performance of our pre-trained models across three scenarios, allowing a direct comparison with human expert evaluations. Each clip within our test set was assigned a distinct numerical identifier. These identifiers and the corresponding ground-truth (GT) labels were documented in an Excel spreadsheet for recording outputs and analysis. To ensure the integrity of our diagnostic assessment, each expert conducted their evaluations independently.

The algorithm performance was assessed on each LUS clip to simulate clinical evaluation practices, similar to how clinicians assess entire videos during diagnosis. For algorithmic assessment, the conversion from frames to video clip analysis adopts a Simple Majority Voting scheme (SVE) to aggregate individual frame predictions into a singular diagnosis for each video clip (37). This transition to video clip analysis compiles predictions from individual frames into a single diagnosis for each video clip. The class with the highest number of predictions is taken as the output prediction for the entire clip. To qualify as a single-video diagnosis, the model must identify healthy or IS frames that comprise more than 50% of the video's total frames, ensuring that they represent a significant portion of the video frames. Upon completion of these assessments, the diagnostic results from each expert were cross-referenced with the GT labels. This comparative analysis enabled us to determine the accuracy of each expert's predictions by identifying correct and false predictions. All developed DL models were evaluated and compared with our clinical experts, using accuracy, sensitivity, and specificity as performance metrics. In addition, a receiver operating characteristic (ROC) curve, which is created by plotting the true positive rate (TPR) against the false positive rate (FPR), was used in the comparative analysis to discern the strengths and limitations of DL models in IS detection in comparison with our medical experts.

## 3 Results

### 3.1 Model performance metrics

**Figure 9:**
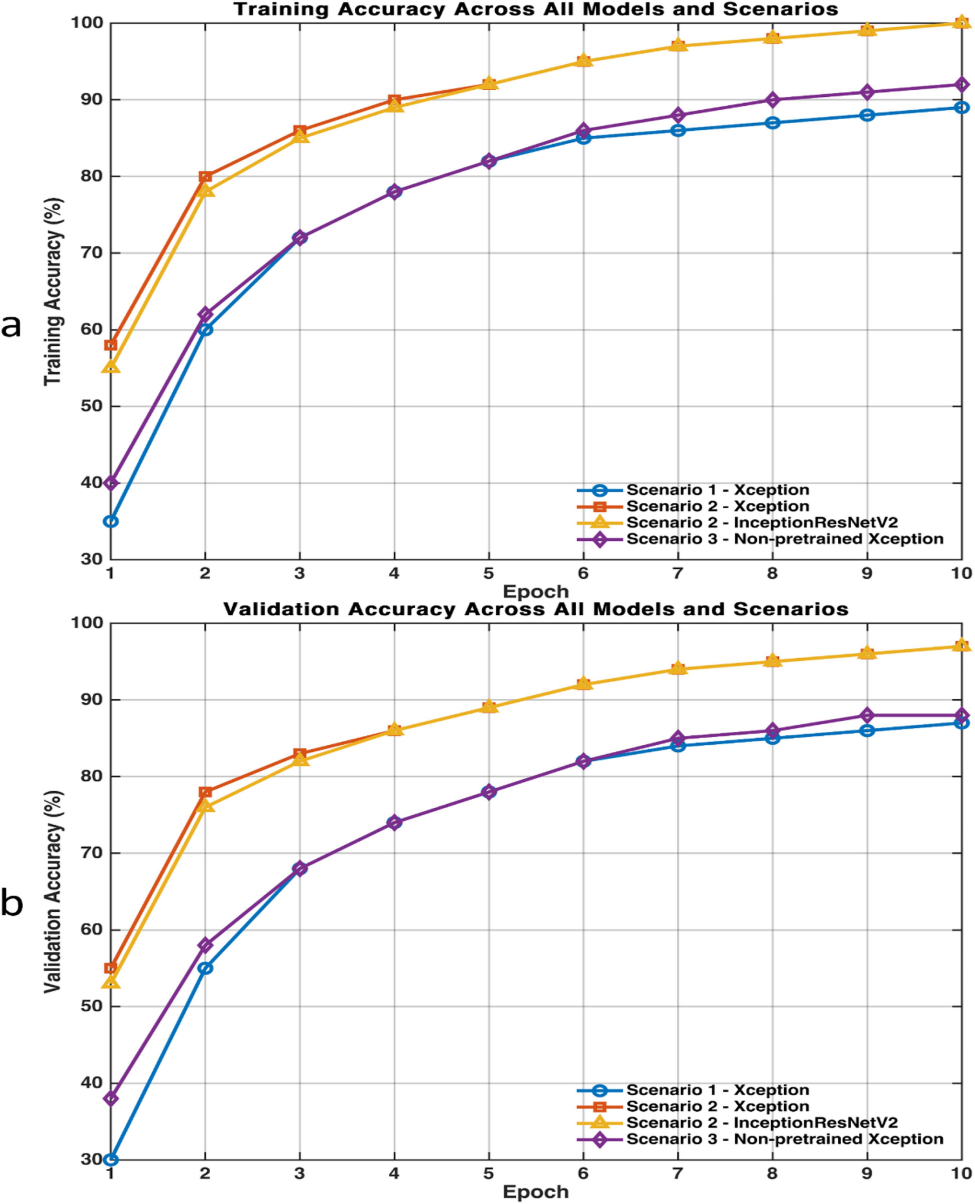
Learning curves showing **(a)** training and **(b)** validation accuracies across all models among scenarios. The close alignment between the training and the validation curves indicates smooth convergence and minimal overfitting.

To assess training stability of the DL models, learning curves for training and validation accuracies were analysed across all models and scenarios (Figure 9). The results showed a consistent upward trend in training accuracy with increasing epochs, confirming effective learning progression. Models in Scenario 2 (Xception and InceptionResNetV2) converged rapidly within the first few epochs and achieved stable performance by the final epoch, reaching approximately 99% and 95% rates for training and validation accuracies, respectively. In comparison, the models in Scenarios 1 and 3 exhibited a slower convergence pattern and slightly lower final accuracy rates, plateauing near 90%. The minimal gap between the training and the validation curves demonstrates stable learning behaviour and suggests limited overfitting.

The performance of the trained models across the various scenarios was evaluated on the test set using accuracy, precision, recall, and F-1 score. The test set consisted of 2,060 LUS frames extracted from 16 LUS clips (eight healthy and eight non-healthy, with two clips per patient). The GT label used to assess the performance of the DL models is the label corresponding to the whole video. This means that a single label representing the overall LUS video classification is assigned to each video frame, against which the model's predictions for individual frames are evaluated. Table 2 shows that both models of Scenario 2 (i.e., pre-trained models with filtered training data) outperformed those of Scenario 1 and 3 in terms of accuracy, precision, recall, and F1 score. The Xception model in Scenario 2 achieved a higher accuracy rate (95.9%) and higher precision and recall rate (95.8%) than the model in Scenario 1. It also had a higher F1 score of 96.0%. On the other hand, the InceptionResnetV2 model in Scenario 2 achieved an accuracy rate of 95.73% and a specificity, precision, recall, and F-1 score of 95.7%. Lastly, the non-pre-trained model in Scenario 3 achieved a specificity and precision rate of 90.6%, a recall rate of 90.4%, and an F1 score of 90.5%.

**Table 2:** The performance metrics of both models are summarised, based on a frame-based assessment in which the ground truth (GT) is assigned to each frame across the entire clip.

Scenario	Model	Performance metrics				
		Accuracy (%)	Specificity (%)	Precision (%)	Recall (%)	F1 score (%)
Scenario 1 pre-trained	Xception	84.6	85.4	88.1	84.2	86.1
Scenario 2 pre-trained	Xception	**95**.**9**	**96**.**6**	**97**.**1**	**95**.**3**	**96**.**2**
	InceptionResnetV2	**95**.**8**	**95**.**5**	**96**.**1**	**95**.**8**	**96**.**0**
Scenario 3 non-pre-trained	Xception	90.5	88.2	89.7	92.5	91.1

In Scenario 1, all 14,902 frames were included, and no filtering was applied. However, in Scenario 2, only 10,546 frames were included, a filtering technique was applied, and non-healthy frames (without B-lines) from IS were excluded. In Scenario 3, the Scenario 2 filtering criteria were applied, and the model was trained from scratch (non-pre-trained Xception model).

The metrics highlighted in bold indicate the highest performance achieved among DL models.

#### 3.1.1 Frame-based: confusion matrix analysis

**Figure 10:**
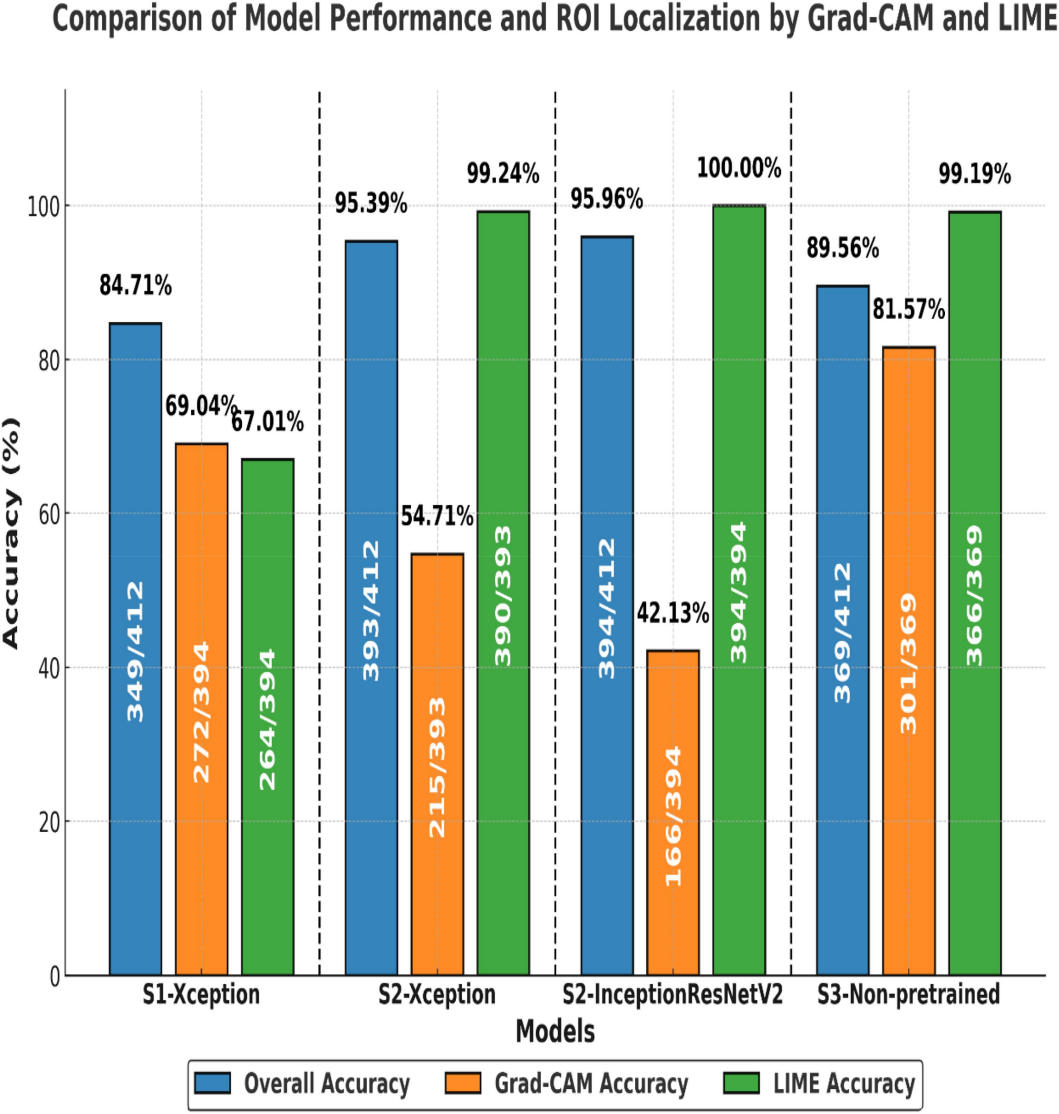
A performance comparison of all models across three measures: overall classification accuracy (blue), Grad-CAM localisation accuracy (orange), and LIME localisation accuracy (green). The results are based on a representative subset of the test set (*n* = 412 frames, sampled from 2,060 total frames). The bars indicate the percentage of correctly classified frames and correctly localised regions of interest (ROIs) identified by Grad-CAM and LIME. Each fraction represents the number of accurate samples out of all evaluated frames. For example, in Scenario 1, the Xception model achieved an 84.7% classification accuracy rate (349/412), while Grad-CAM correctly localised 69.0% (272/394) and LIME 67.0% (264/394) of ROIs.

The results of the four models on the test set (2,060 LUS frames, with 1,115 IS frames and 945 healthy) extracted from 16 clips are presented in Figure 10. The number of false predictions made by the Xception model in Scenario 1 was triple that of the same model in Scenario 2, with 315 frames (highlighted in dark orange) and 84 frames (highlighted in light blue), respectively. These results suggested that the Xception model in Scenario 1 had a significantly higher rate of false predictions compared with the same model in Scenario 2. Overall, the models in Scenario 2 showed a significantly lower proportion of falsely predicted frames compared with Scenario 1 and Scenario 3 (non-pre-trained model). This indicates a greater ability of the Scenario 2 models to accurately distinguish between healthy and non-healthy (IS) frames. Detailed classification performance, including predictions for each clip and the counts of true and false predictions for both healthy and non-healthy frames, is provided in Supplementary Appendix A.

### 3.2 Explainability and interpretability

**Figure 11:**
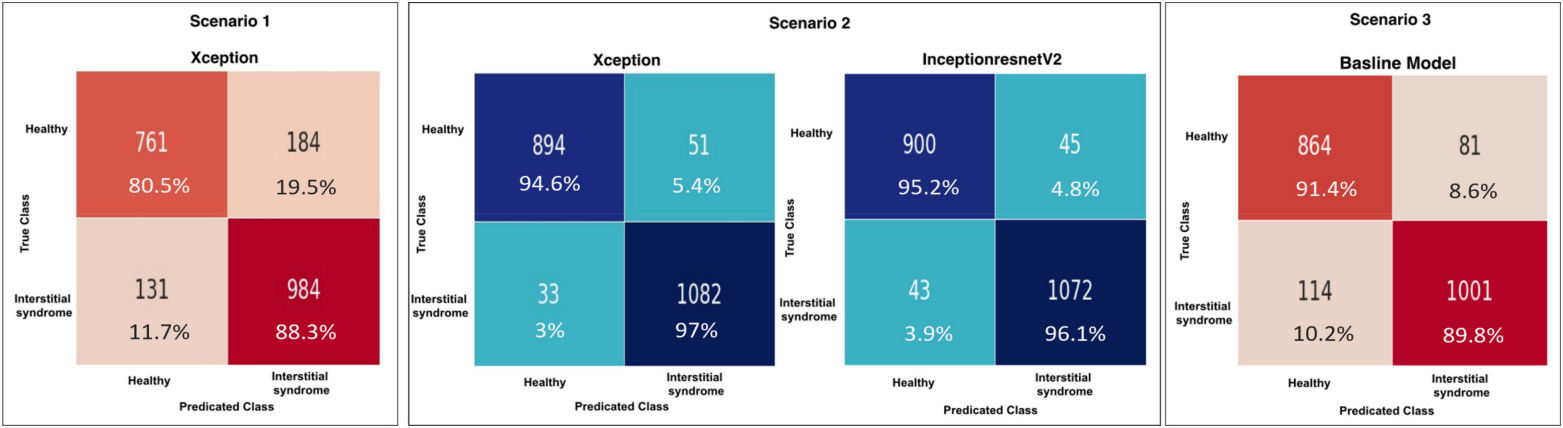
Confusion matrices (CMs) of the developed models on the test dataset using three training techniques. Two color maps are applied consistently across all matrices; the blue tones represent higher accuracy rates (≥90%), while the red tones highlight lower performance (<90%). From left to right, the CMs show Scenario 1 with 315 false predictions, Scenario 2 (Xception model) with 84 false predictions and the InceptionResNetV2 model with 88 false predictions, and Scenario 3 (non-pre-trained model) with 195 false predictions.

A subset of the testing dataset was selected, consisting of 412 frames: 189 healthy (H) frames and 223 non-healthy (IS) frames. A randomly selected subset was used to evaluate the model's performance along with the accuracy of its corresponding Grad-CAM and LIME visualisations, as shown in Figure 11 (with additional details in Supplementary Appendix B). The accuracy of Grad-CAM and LIME was manually assessed by examining how well the highlighted regions aligned with the expected ROI in both IS and healthy cases. For non-healthy cases, the assessment focused on whether the visualisations correctly highlighted existing pathological features (e.g., B-lines). For healthy cases, the evaluation examined whether the visualisation instead emphasised normal lung features, such as A-lines, while avoiding false highlighting of non-existent pathologies such as the background.

**Figure 12:**
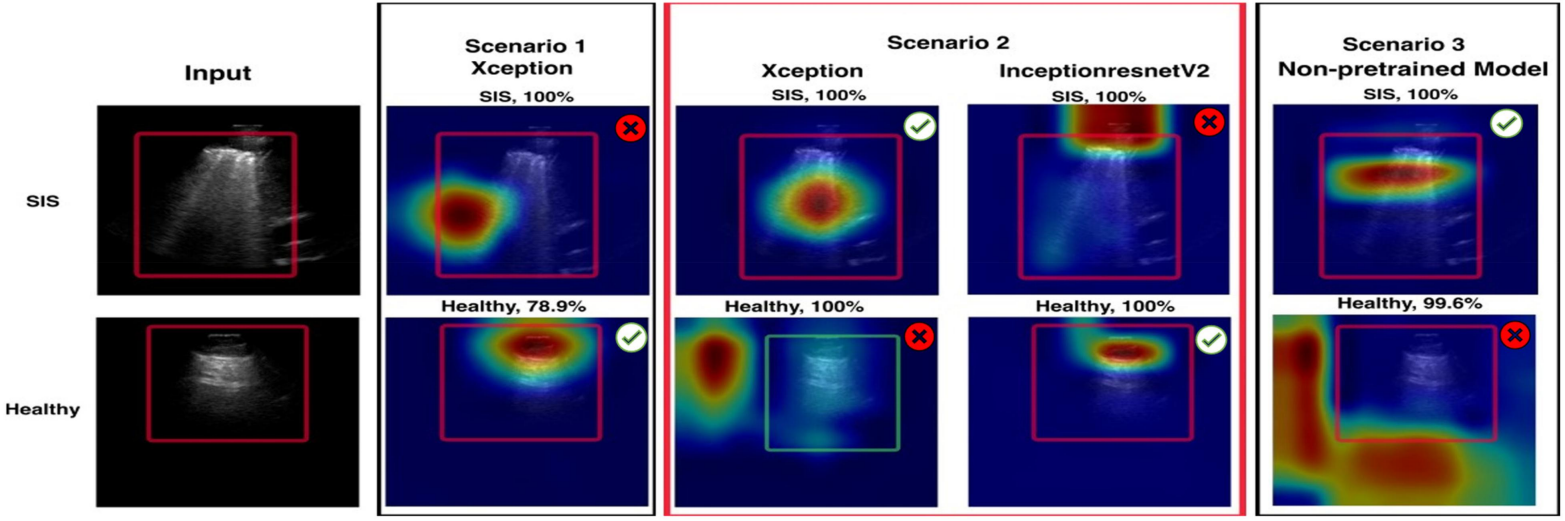
A visualisation of Grad-CAM and confidence values for the LUS frames predicted for SIS and healthy frames for all models. The red box in the input image indicates the intended region of interest (ROI). In each scenario, the images display Grad-CAM with a red box highlighting the ROI. Each image displays Grad-CAM with a red box indicating the intended ROI. A red cross is shown if Grad-CAM does not align with the ROI, and a green checkmark is shown if it does.

Figures 11, 12 summarise model performance and Grad-CAM visualisations for healthy and SIS LUS frames. In the heatmaps, warmer colours (red/orange) represent areas of stronger model activation, whereas cooler colours (green/blue) denote weaker activation. The red box marks the predefined ROI used for evaluation. In healthy examples, the apparent shift in the ROI relative to SIS frames reflects the model's attention to normal pleural lines or A-lines rather than to vertical B-lines.

**Figure 13:**
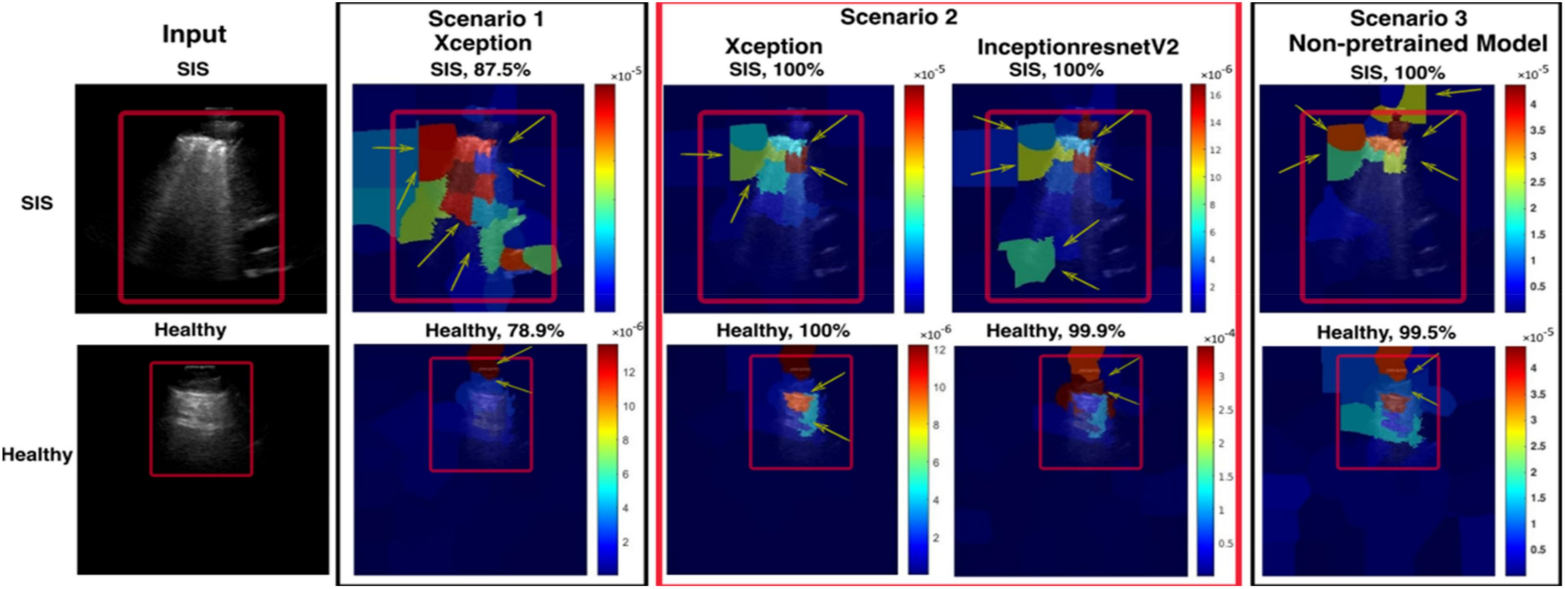
A visualisation of LIME and confidence values for LUS frames predicted as SIS and healthy frames for all models. For the SIS sample, the Scenario *1* model focuses *on* multiple areas*,* shown *as a* diffuse pattern*,* marked in green*,* red*,* and blue. In contrast, the Scenario *2* model focuses on IS features (yellow arrows) within the intended ROI (marked with the red box on the input image). For the healthy sample, the Scenario 1 model focuses on multiple areas, marked in blue and red. In contrast, Scenario 2 focuses on IS features (yellow arrows) within the intended ROI, marked with red, orange, and blue colours.

The Xception model in Scenario 1 achieved an overall accuracy rate of 84.71%, correctly classifying 79.89% of healthy frames and 88.79% of non-healthy frames. Grad-CAM, as shown in Figure 12, highlighted relevant areas in 83.44% of healthy and 73.74% of SIS frames, while LIME, as shown in Figure 13, provided a better accuracy rate for healthy frames (100%) but only a rate of 57.07% for SIS frames. In Scenario 2, Xception performed better with a 95.39% accuracy rate, but Grad-CAM, as shown in Figure 12, failed to identify ROIs in healthy frames (0%), while excelling in SIS frames (100%). As shown in Figure 12, LIME demonstrated more consistency, with a 98.31% accuracy rate for healthy and 100% rate for SIS frames. The InceptionResNetV2 model in Scenario 2 achieved the highest accuracy rate (95.96%), effectively identifying healthy (95.24%) and SIS (95.96%) frames. Grad-CAM, as shown in Figure 12, performed well for healthy frames (92.22%) but failed for SIS (0%), while LIME, as shown in Figure 13, correctly identified all frames (100%). Scenario 3 (non-pre-trained) reached an 89.56% accuracy rate with Grad-CAM, as shown in Figure 12, inconsistently localising healthy frames (59.76%) but excelling in SIS frames (100%). In contrast, as shown in Figure 13, LIME remained highly reliable for both classes (98.22% and 100%). More details can be found in Supplementary Appendix B.

### 3.3 False predictions

All the false predictions of the Xception model in Scenario 2 were re-evaluated because of its superior performance in terms of accuracy among the four models evaluated in Scenario 1, Scenario 2, and Scenario 3. This thorough evaluation aimed not only to capture errors in the model but also to rigorously assess whether the identified predictions were truly false, thereby enhancing our understanding of the model's diagnostic reliability. A total of 84 frames were reviewed by our expert (MS), including 33 false positives and 51 false negatives identified by the model.

**Figure 14:**
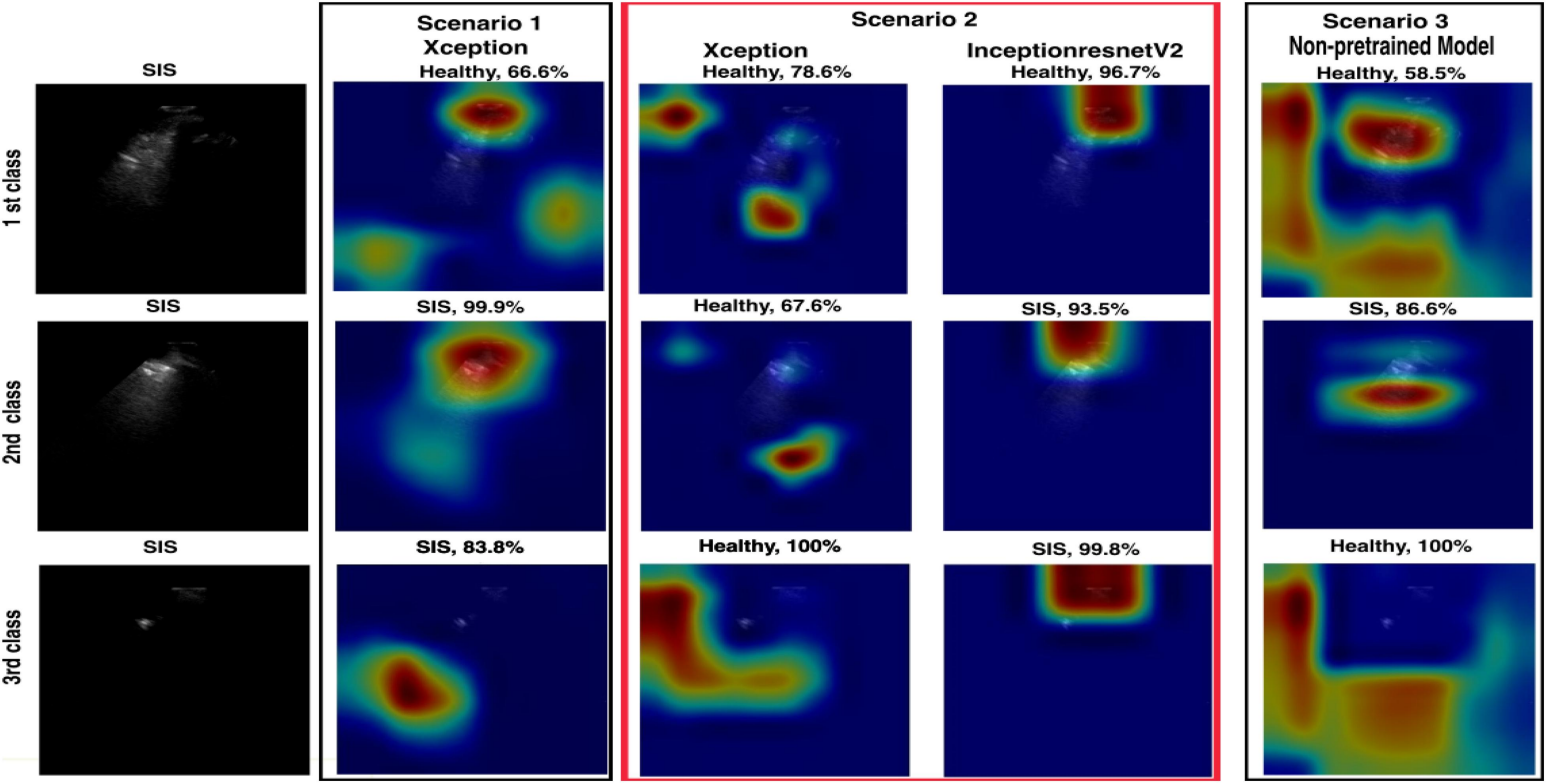
A comparison of models in the three scenarios where the Xception model in Scenario 2 is more confident in predicting healthy frames for those mislabelled as SIS, with higher confidence values (67.6%–100%).

For the false positive frames (33 out of 1,115 = 2.96%) where the Xception model in Scenario 2 predicted “healthy,” the expert re-evaluated 33 frames. The clinical expert categorised these frames into three classes, as shown in Figure 14. In the first class, 22 of the 33 frames (77.67%) emerged. Our clinicians classified the frames as healthy because of either the absence of B-lines or limited visibility, which matches the Xception model's prediction in Scenario 2. Only 1 of the 33 frames (3.03%) exhibited potential B-lines in the second class. In the third class, 10 of the 33 frames (30.3%) were classified as non-diagnostic or marked with limited visibility because of the shadowing caused by ribs.

**Figure 15:**
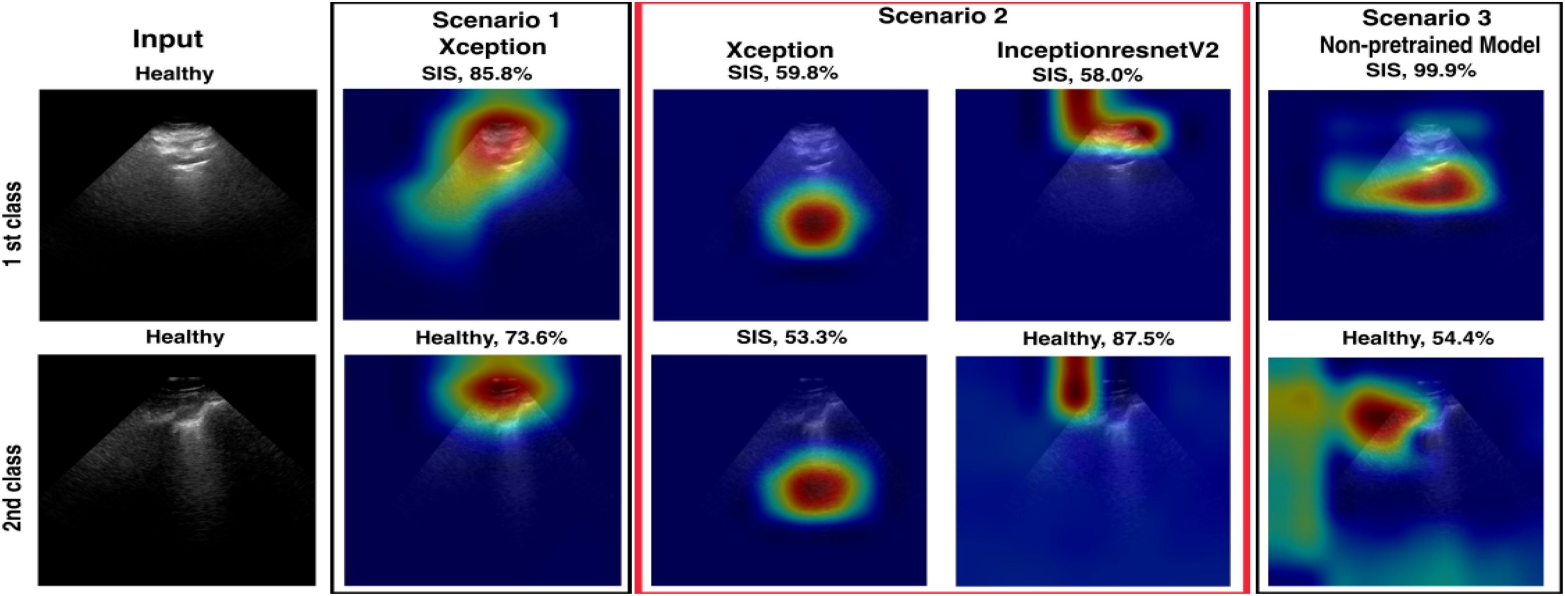
A comparison of Scenarios 1, 2, and 3, where the Xception model in Scenario 2 is mislabelled in the upper example (first class) with a low confidence value and correctly predicted in the lower example (2nd class).

In the case of false negatives, where the model incorrectly identified frames as “IS,” the expert re-evaluated 51 frames (51 out of 945 frames = 5.4%). For the first class, the clinicians determined 11 of the 51 frames (21.57%) to be IS, indicating that the model's predictions were incorrect. The remaining 40 of the 51 frames (78.43%) were considered healthy, as there was no evidence of B-lines. Figure 15 shows examples along with each of the two classes.

Upon re-evaluating the false prediction frames (84 frames) of the Xception model in Scenario 2, the model correctly predicted false positives, with a 22% (out of 33) success rate. However, the model correctly predicted only 11 out of 51 false negatives. This demonstrates the importance of analysing videos at the frame level to better understand how the algorithm performs. Also, not all frames in a video labelled as IS necessarily show IS features, which should be considered in the evaluation.

### 3.4 Evaluation of fusion ML classifiers

Multiple ML classifiers were trained using features extracted from the three models (second and third scenarios) previously mentioned.

#### 3.4.1 ML classifiers' performance metrics

The performance of the best ML classifier in the various fusion processes was evaluated in terms of accuracy, precision, recall, and F1 score. As shown in Table 3, both the binary GLM logistic regression (F1) and the neural network (F2) models accurately predicted 98.2% of the LUS frames. The binary GLM logistic regression model achieved an F1 score of 98.2%, with a precision rate of 97.3% and a recall rate of 99.2%. The neural network model achieved a slightly higher F1 score of 98.3%, with a precision rate of 98.2% and a recall rate of 98.4%. The KNN model in F3 achieved an accuracy rate of 95.8%, a precision rate of 94.9%, a recall rate of 97.3%, and an F1 score of 96.1%. Lastly, the neural network model in F4 achieved an accuracy rate of 97.0%, with a specificity rate of 96.7%, a precision rate of 97.2%, a recall rate of 97.0%, and an F1 score of 97.1%. Overall, both ML classifiers in F1 and F2 outperformed ML classifiers in F3 and F4 in terms of accuracy, precision, recall, and F1 score.

**Table 3:** The summary performance metrics of the developed models on the test dataset (2,060 frames).

ML classifiers		Performance metrics				
		Accuracy (%)	Specificity (%)	Precision (%)	Recall (%)	F1 score (%)
F1	Binary GLM Logistic R	**98**.**2**	**97**.**0**	**97**.**3**	**99**.**2**	**98**.**2**
F2	Neural network	**98**.**2**	**97**.**9**	**98**.**2**	**98**.**4**	**98**.**3**
F3	KNN	95.8	94.1	94.9	97.3	96.1
F4	Neural network	97.0	96.7	97.2	97.0	97.1

F1 is a feature fusion of all models mentioned in Scenarios 2 and 3 (Xception, InceptionResnetV2, and the non-pre-trained model). F2 is a feature fusion between the two best models from Scenario 2. F3 and F4 are feature fusions between the non-pre-trained model and Xception in Scenario 2 and the non-pre-trained model and InceptionResnetV2 in Scenario 2, respectively.

The metrics highlighted in bold indicate the highest performance achieved among the ML models.

#### 3.4.2 ML classifiers' confusion matrix (frame-based assessment)

**Figure 16:**
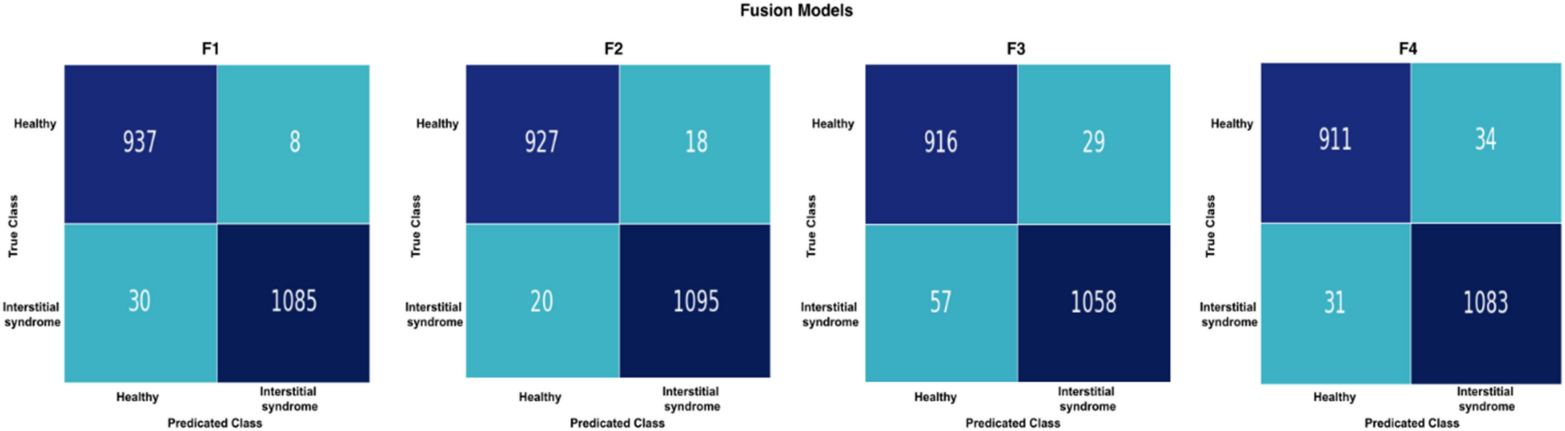
A confusion matrix of the fusion models on the test set (2,060 LUS frames) with four different fusion processes: left, F1 and F2 both with 38 false predictions; right, F3 with 86 false predictions and F4 with 88 false predictions.

To further evaluate the performance of the ML classifiers, confusion matrices are presented in Figure 16. F1 and F2 had the same number of false predictions, with 38 frames each (highlighted in light blue). In contrast, F3 and F4 had much higher rates of false predictions, with 86 and 65 frames, respectively (highlighted in light green). Overall, these results indicated that F3 and F4 were less accurate than F1 and F2 in classifying the test data. This improved performance indicated a greater capacity of the fused models in F2 to accurately differentiate between non-healthy (IS) and healthy frames. More detailed classification performance, including predictions for each clip, the number of LUS frames, and the number of true and false predictions for both healthy and non-healthy frames, is provided in Supplementary Appendix C. In addition, Supplementary Appendix D provides an example comparison of confidence values in Scenarios 1, 2, and 3.

### 3.5 The experts compared with our models (video-based assessment)

**Figure 17:**
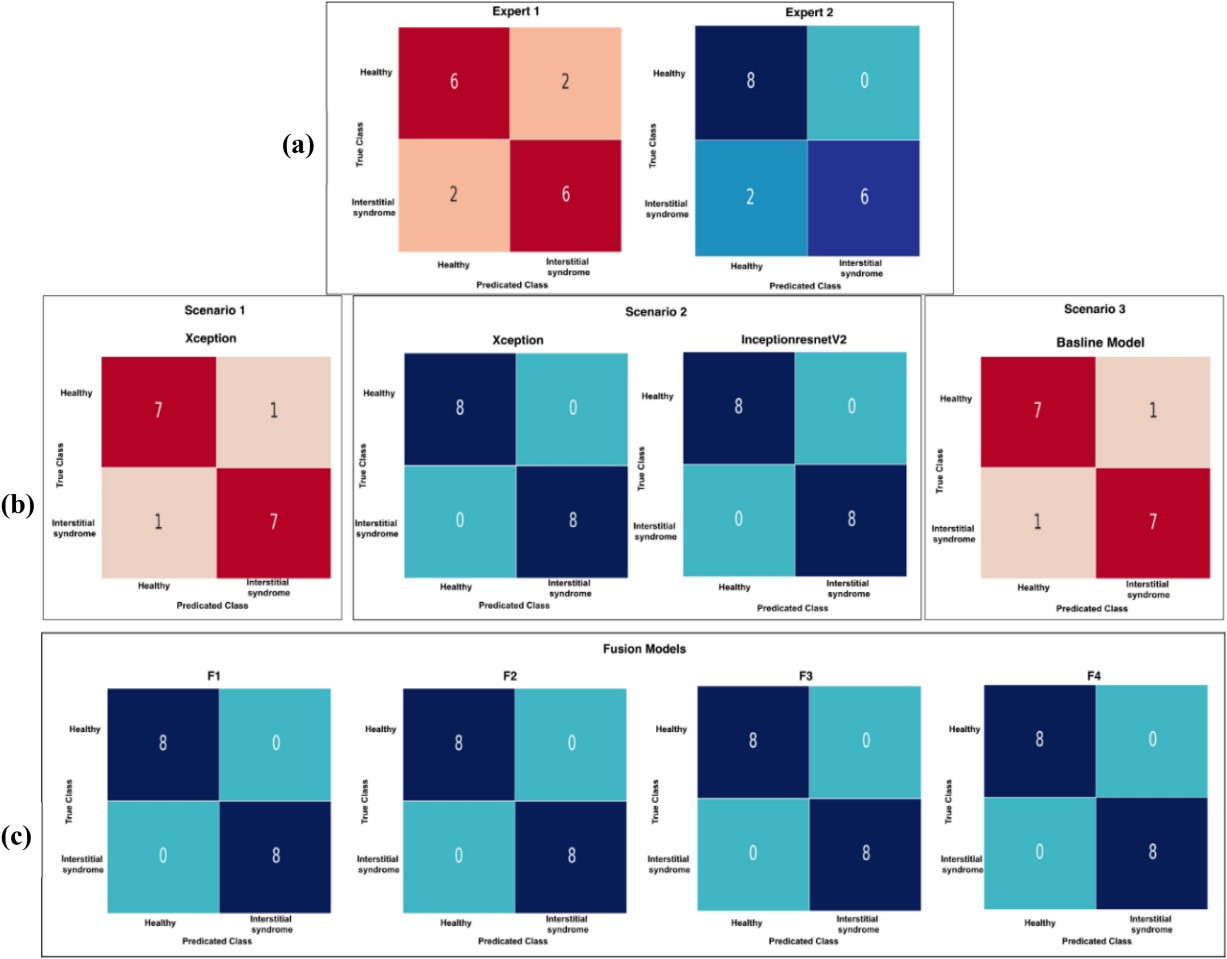
A confusion matrix (CM) of our experts and developed models on 16 LUS clips (eight healthy and eight IS). **(a)** The CM of our first expert with four false predictions, while our second expert shows only two false predictions. **(b)** The CM for all developed models in Scenarios 1, 2, and 3. In Scenario 1, both Xception and InceptionResNetV2 models demonstrate perfect classification for all clips. In Scenario 3, the non-pre-trained model shows two false predictions. **(c)** The CM for all fused models (F1, F2, F3, and F4), where all models achieve perfect classification with no false predictions.

All developed AI and ML models were evaluated in terms of true and false positives and negatives, as shown in Figure 17, following the methodology described in Section 2.6. The GT label used to assess the performance of the DL models compared with our experts is the label that matches the entire LUS clip label. Our first expert (MS) identified 75% (12 clips) of the labelled cases within the sample test subset (16 clips) as correct predictions and 25% (four clips) as false predictions of the labelled cases. In contrast, our second expert (CE) identified 88% (14 clips) of the labelled cases within the sample test subset (16 clips) as correct predictions and 12% (two clips) as false predictions of the labelled cases. Among all developed models, both Scenario 2 and fusion models F1, F2, F3, and F4 predicted 100% of the LUS clips with the best accuracy rates. They identified 100% (16 clips) of the labelled cases within the sample test subset (16 clips) as correct predictions with no false predictions. This gives these models an F1 score of 100%, a precision rate of 100%, and a recall rate of 100%.

**Figure 18:**
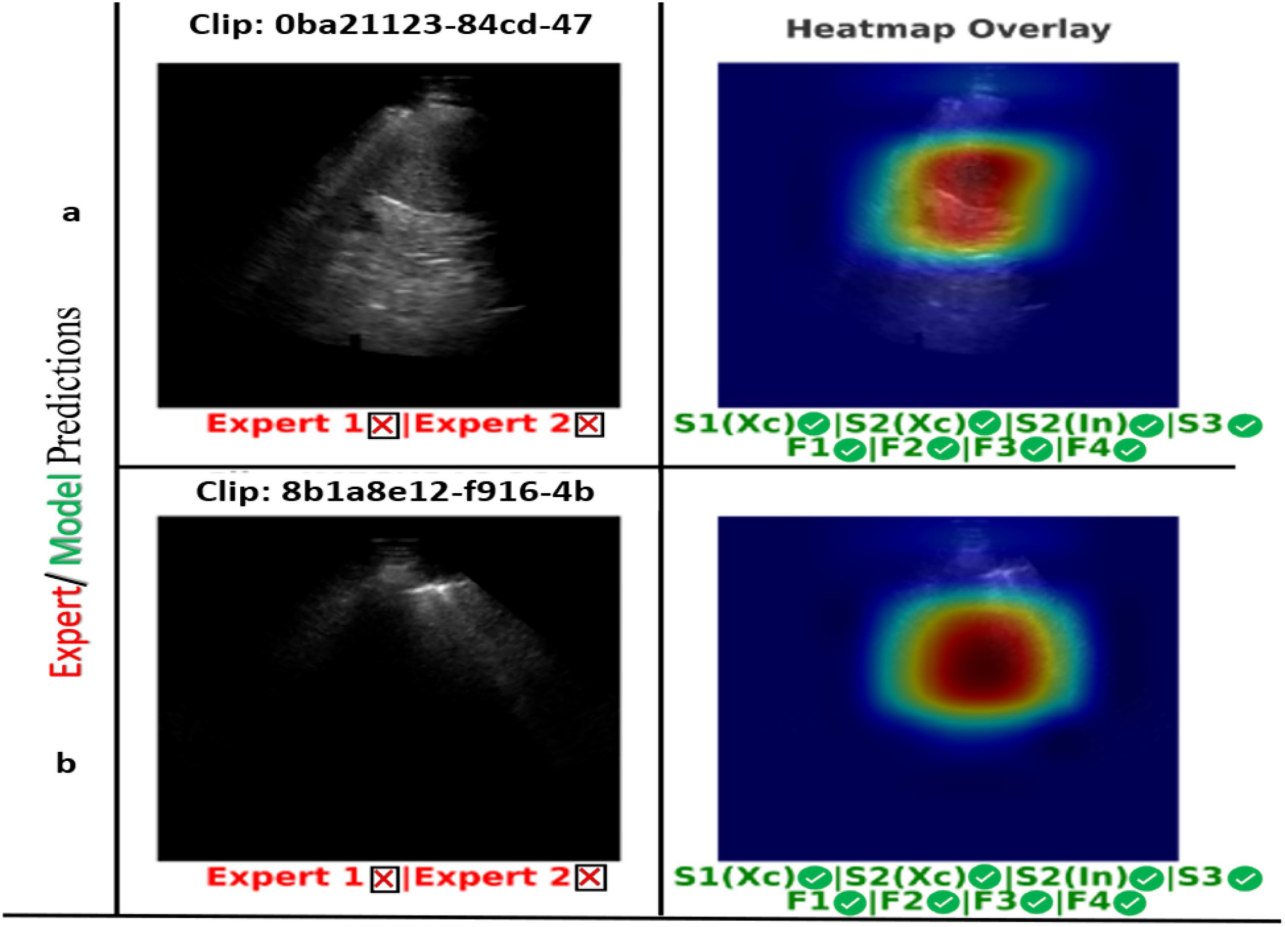
The two LUS clips demonstrating diagnostic disagreement between Expert 1 and Expert 2 relative to the GT (red “×”) are shown on the left **(a and b)**. The corresponding heatmaps for Scenario 2 (Xception model), with correct model predictions (green “✓”) displayed on the right.

**Figure 19:**
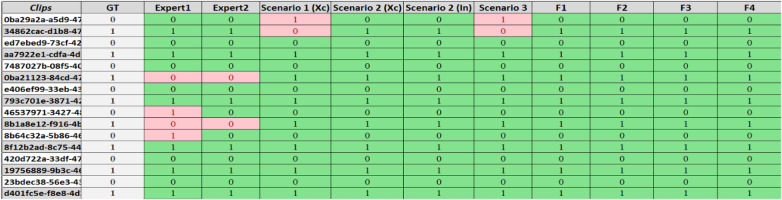
This spreadsheet shows the evaluation output for LUS clip predictions as determined by our experts alongside developed models. It shows a comparative analysis of predictions across 16 clips, with the ground truth (GT) labels for IS and healthy states as the benchmark. Predictive outcomes are marked “1” for IS and “0” for healthy clips, with false predictions highlighted in pink.

The DL models in Scenario 1 and Scenario 3 identified 88% (14 clips) of the labelled cases within the sample test subset (16 clips) as correct predictions and two as false predictions, as shown in Figure 18. Upon reviewing the two LUS clips in Figure 18, it was found that both experts faced challenges in identifying IS cases, and these have been highlighted. In Clip (a), experts classified the clip as a solid organ with peripheral B-lines but did not confirm the presence of three B-lines. Clip (b) also shows a mixed pattern with scattered B-lines, complicating the verification of the presence of three B-lines. Both cases show subjectivity in B-line interpretation and quantification, as experts struggled to distinguish artefacts from true pathology. Figure 19 shows a spreadsheet capturing the evaluation process and outcomes of LUS clip predictions performed by our experts and all developed models on the set of 16 clips. Each column under the experts and models represents their predictions for the clips, with “1” indicating an IS prediction and “0” representing a healthy clip. The cells highlighted in pink mark the instances where a false prediction was recorded by the corresponding expert or developed model. Overall, both ML classifiers (F1 and F2) and models in Scenario 2 outperformed our expert performance and other developed AI and ML models in terms of accuracy, precision, recall, and F1 score. A more detailed display of the performance of AI and ML models is provided in Table 4.

**Table 4:** The performance metrics of the developed models on 16 LUS clips (8 healthy and 8 IS).

Expert/Scenario/ Fusion Models	Model	Performance metrics				
		Accuracy (%)	Specificity (%)	Precision (%)	Recall (%)	F1 score (%)
Expert 1		75	75	75	75	75
Expert 2		87.5	100	100	75	85.71
Scenario 1	Xception	87.5	87.5	87.5	87.5	87.5
Scenario 2	Xception	**100**	**100**	**100**	**100**	**100**
	InceptionResnetV2	**100**	**100**	**100**	**100**	**100**
Scenario 3	Non-pre-trained model	87.5	87.5	87.5	87.5	87.5
F1	Binary GLM Logistic R	**100**	**100**	**100**	**100**	**100**
F2	Neural network	**100**	**100**	**100**	**100**	**100**
F3	KNN	**100**	**100**	**100**	**100**	**100**
F4	Neural network	**100**	**100**	**100**	**100**	**100**

The metrics highlighted in bold indicate the highest performance achieved among the ML and the DL models.

**Figure 20:**
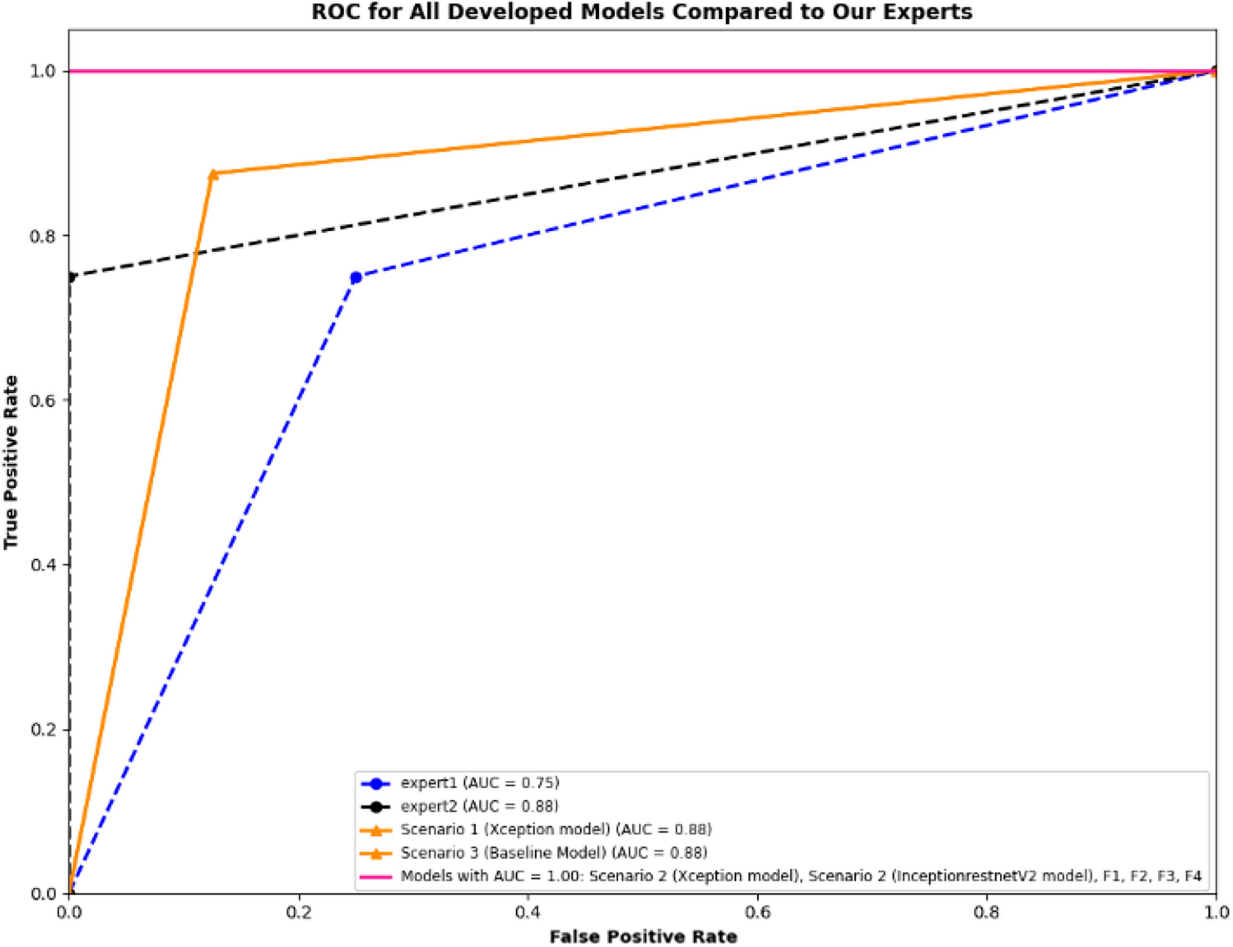
The performance of developed models and our clinical experts on a set test (*n* = 16). The dotted lines represent the performance of clinical experts (blue for Expert 1 AUC = 0.75 and black for Expert 2 AUC = 0.88). The triangular markers with solid lines represent the performance of deep learning (DL) models in Scenarios 1 and 3 (AUC = 0.88). The pink solid line shows the superior performance of the developed models with AUC = 1.00, including both Scenario 2 models (Xception and InceptionResnetV2) and fusion models F1–F4.

Furthermore, Figure 20 illustrates the ROC curve, which shows the performance of our experts and developed models via TPR and FPR. The ROC curve shows that the fused F1, F2, F3, F4, and Scenario 2 models significantly outperform our experts and other developed models. However, it is important to note that this evaluation is based on a small subset of videos.

## 4 Discussion

This work explores the value of using transfer learning CNN models and fusion techniques to enhance the performance of deep learning models for classification tasks, specifically in identifying B-lines and Interstitial Syndrome in LUS frames. In addition, the work evaluates how applying filtering techniques during the training process can improve accuracy and reduce disagreement rates.

The fused models in Feature Fusion 1 (F1) and Feature Fusion 2 (F2) models, where the first (F1) combined all models from Scenario 2 (Xception, InceptionResNetV2, and the non-pre-trained model in Scenario 3) and the latter (F2) combined the best-performing models from Scenario 2, remarkably display substantial enhancements across a spectrum of performance metrics compared with all developed models, as shown in Section 3.5. This substantial improvement can be attributed to the strategic use of filtering techniques during the training phase in Scenario 2 and the fusion technique used.

The fused models in (F1 and F2) demonstrate a notable surge in the accuracy rate (98.2%) compared with other models. This significant improvement indicates that the applied filtering techniques have resulted in more precise overall predictions. In addition, the fusion models exceed accuracy rates compared with the best individual models in Scenario 2 (95.9% and 95.8%).

Importantly, the fused models in (F1 and F2) showed a notable reduction in false predictions relative to the individual DL models in Scenario 2. This reduction provides tangible evidence that the incorporated filtering techniques and combined features into fused models have effectively mitigated the model's tendency to misclassify healthy instances as IS and vice versa.

A comparison between the fused models, F1 and F2, and individual models in Scenario 2 revealed a decrease in false predictions (including false negatives and positives) within the fused models. F1 and F2 models mislabelled only 38 frames (1.8%), a significant improvement compared with the 84 frames (4.05%) mislabelled by the Xception model in Scenario 2. In addition, within the individual models in Scenarios 1, 2, and 3, the Grad-CAM and LIME visualisations supported our method of excluding healthy frames from IS frames during training of the Xception model in Scenario 2, even with the small dataset used, compared with the Xception model in Scenario 1. This method significantly impacted the observed performance differences between the two models.

A notable observation in this study involves the Grad-CAM visualisation, as it often highlights areas outside the ROI. As shown in Section 3.2 (Figure 12), these visualisations may inconsistently align with clinically relevant features, thereby limiting their explainability and clinical utility. In contrast, LIME visualisations offer more precise and interpretable explanations that closely match the ROI, making them more useful for clinical assessments (Figure 13). A comparison of Xception and InceptionResnetV2 in Scenario 2 and the non-pre-trained model in Scenario 3, using LIME, on a subset of the test set (412 out of 2,060 frames), highlighted the efficacy of our suggested transfer learning technique with 390/390 (99.24%) and 394/394 (100%), respectively. Overall, with the same dataset used for the training, both models in Scenario 2 performed better than the non-pre-trained models trained from scratch.

As noted in our previous work (4, 6), excluding healthy frames from the non-healthy class may have had a discernible impact on the performance of the AI model. In Scenarios 2 and 3, by eliminating the potential bias caused by healthy frames within clips showing IS features, the models became more adept at distinguishing between IS and healthy frames, resulting in higher accuracy and precision. In Scenario 1, the Xception model, which did not involve any frame exclusion, had its accuracy rate set at 84.6%. In contrast, in Scenario 2, the Xception model, which applied filtering techniques, substantially improved the overall accuracy rate to 95.9%. This emphasised the substantial enhancement conferred by applying filtering to the training dataset instead of utilising all frames in the training process.

Furthermore, upon evaluation of a subset of 16 clips in Section 3.5, the result showed that the fused models, F1, F2, F3, F4, and Scenario 2 models, outperformed the performance of our experts in classifying healthy and IS LUS clips. This suggests that developed AI and ML models have a high level of accuracy, precision, recall, and F1 score in detecting healthy and IS from LUS frames, showing higher agreement with clinical diagnoses compared with our medical experts. Expert 1 (MS) had a high false positive rate (25%; four clips), indicating challenges in distinguishing between healthy and IS LUS clips during the video-level assessment. Comparably, Expert 2 (CE) showed high diagnostic accuracy, with a reduced rate of false predictions, with only 12.5% of the clips (two out of 16). This result highlights that experts conducting *post hoc* video assessments may encounter additional challenges because of the absence of real-time evaluation, which usually aids in diagnosis. Also, in our comprehensive assessment of detecting IS, the case of diagnostic disagreement emerged, highlighting the challenges in LUS interpretation. Our clinical experts from Melbourne (DC and XC) initially classified those LUS clips as indicative of a healthy or IS lung condition. This initial assessment was based on IS criteria from the international evidence-based recommendations (28) and their clinical expertise. It reflects a view at the time of their acquisition and is based on multiple zones of the patient's lung.

Upon re-assessment of the two mislabelled LUS clips discussed in Section 3.6, our experts, reviewing the same clip for each patient, identified challenges related to consistent video interpretation. This highlights the complexity of diagnosing LUS clips, particularly when the field of view is limited, image quality is poor, and there is no prior knowledge of patient history. This disagreement among experts shows the subjectivity and variability that define the interpretation of LUS clips, particularly with artefacts related to B-lines. Such variability arises from the diverse interpretations by experts regarding the origin of B-lines—whether they emanate from the pleural line or not—and the quantification of these B-lines. Contributing to these discrepancies are factors such as a constrained field of view and the challenges posed by poor-quality LUS clips. These elements highlight the complexities of LUS analysis. In contrast, developed AI and ML models accurately predicted most test clips, often surpassing our medical experts on complex cases.

This project is part of the ongoing research collaboration between Melbourne University and QUT researchers in the field of LUS pathology evaluation using AI tools. Our collaborative research team has recently proposed a fully automated LUS evaluation for lung pathologies, including pleural effusion, atelectasis (collapse), consolidation, and pneumothorax. As part of this collaboration, a study by Tsai et al. (6) achieved a 92% accuracy rate in classifying pleural effusion using a DL model consisting of a Regularised Spatial Transformer Network. A follow-up study by Durrani et al. (4) demonstrated DL's potential for diagnosing pulmonary consolidation or collapse with an 89% accuracy rate. Our current work has resulted in the development of the best-trained ML model that achieved a test accuracy rate of 98.2%. This result demonstrates for the first time the use of pre-trained CNNs with a feature extraction and fusion method to develop a diagnostic tool for IS screening in LUS frames. However, all models in this study have been trained and tested on a frame-level basis, meaning that their ability to generalise across entire LUS videos remains questionable and largely depends on how many frames within LUS videos are classified as non-healthy. Our next step will focus on exploring AI capabilities to analyse entire LUS video clips, particularly those containing mixed pathologies, enabling a more robust and clinically relevant real-time assessment of lung pathologies.

A notable limitation of this study is that we used only a small test set (16 clips) to evaluate the performance of our experts compared with the developed models. This may not be representative of the general population or of the different settings in which LUS imaging is performed. Furthermore, only two experts evaluated these test clips. Therefore, further studies are needed to test the performance of our experts on larger and more diverse datasets of LUS with the involvement of additional LUS experts.

Another limitation of this study arises from using DL models that were initially pre-trained on general-purpose image classification using natural images (out-of-domain dataset). We then retrained these models using our specific LUS dataset (the target dataset). As a result of the inherent differences between the original dataset (comprising natural images) and our target dataset (comprising LUS images), DL models may produce visualisations where discrepancies may be observed between Grad-CAM and LIME. As shown in Section 3.2, LIME demonstrates superior interpretability for both healthy and non-healthy testing LUS frames, showing precise localisation of the ROI across most DL models. In contrast, Grad-CAM shows inconsistencies, particularly in ROIs of healthy examples with the Xception model in Scenario 2 (Figure 12). Nevertheless, the consistent classification performance across models and datasets indicates that these visualisation differences do not imply exploitation of spurious correlations. These findings highlight the need to improve AI explainability methods such as Grad-CAM and LIME to ensure more consistent and accurate visualisation of reliable feature attribution, ultimately enhancing trust in AI-assisted clinical decision-making. In our future work, we plan to enhance the model's performance by re-training it using a larger dataset and categorising it into multiple classes to identify multiple LUS pathologies.

A further limitation of this study is that the proposed framework was designed for binary classification (IS vs. healthy), without distinguishing between specific causes of Interstitial Syndrome, such as pulmonary oedema, ILD, or pneumonia. Differentiating among these pathologies is clinically relevant because management and prognosis differ substantially. Future work will focus on developing and validating a multi-class classification approach to enable the model to identify individual IS aetiologies, thereby enhancing its clinical applicability.

Another area for improvement is that labelled LUS clips used in this study were derived from medical reports, which were generated based on video-based labelling. For future work, we aim to access a larger dataset and train the model using full LUS videos rather than individual frames for both training and testing. This approach will allow the model to capture temporal patterns and contextual information, potentially improving its performance. In addition, testing the AI model on videos more closely mimics how clinicians interpret LUD in real-world settings, as they assess dynamic changes rather than isolated frames.

The high diagnostic performance demonstrated in this study supports the potential for incorporating deep learning–based LUS analysis into POCUS workflows. For practical deployment, future versions of this system could be embedded in portable ultrasound devices or cloud-connected platforms to provide real-time diagnostic feedback during scanning. Successful integration will require optimisation for processing speed, standardisation of LUS acquisition protocols, and a clinician-friendly interface that displays AI-derived overlays alongside conventional ultrasound images. In addition, prospective validation in larger and more diverse patient cohorts will be essential to confirm generalisability and compliance with clinical and regulatory standards.

## 5 Conclusion and future work

The pre-trained models utilised in this study functioned effectively as an automated tool for identifying IS in LUS video frames. Furthermore, fusion models from features extracted from those models outperformed the individual DL modes in terms of accuracy.

Future work in this area could further enhance the applicability and reliability of the proposed CNN models using transfer learning and feature fusion for other lung diseases. This includes expanding the dataset size and diversity, which could help validate the model's generalizability across different patient populations, diseases, and imaging conditions. In addition, investigating the model's performance in distinguishing between different types of Interstitial Syndrome could provide valuable insights into its potential clinical utility for other LUS disorders. In conclusion, future work needs to focus on expanding the dataset and performing comprehensive validation across different LUS datasets. However, the current study shows encouraging results in IS screening using pre-trained models and LUS frames. This advancement in deep learning techniques will contribute to the establishment of an accurate, reliable, and clinically valuable tool for diagnosing and managing LUS disorders.

## Data Availability

The datasets presented in this article are not readily available because they are subject to institutional data-sharing restrictions at Queensland University of Technology (QUT). Requests to access the datasets should be directed to the corresponding author. Please let us know if any further information or clarification is required.
