# Supplementary material for: Convolutional automatic identification of B-lines and interstitial syndrome in lung ultrasound images using pre-trained neural networks with feature fusion
Source: Front Digit Health. 2026 Jan 19;7:1632376. doi: 10.3389/fdgth.2025.1632376 (PMC12862092; doi:10.3389/fdgth.2025.1632376)

# **Appendix** A

Figures 1, 2, 3, and 4 show a comprehensive overview of the models' performance on the test set, 16 LUS clips, with a total frame number of 2060 (1115 IS frames and 945 healthy). The figures show the model classification performance for each clip, including the count of LUS frames and true and false predictions for healthy and non-healthy frames. For example, in case number 2-2 from Fig 1-b, which is labelled as IS, the Xception model in Scenario 1 accurately identified 35 out of 120 frames as non-healthy (IS) while incorrectly predicting 85 frames as healthy. In contrast, considering the same case number, the Xception model in Scenario 2, as indicated in Fig 2-b, performed better by correctly predicting 112 out of 120 frames as non-healthy (IS) and only making 8 incorrect predictions of healthy frames. Furthermore, according to Fig 3-b, the InceptionrestnetV2 model in Scenario 2 correctly predicted 119 out of 120 frames as non-healthy (IS) and only made 1 incorrect prediction in the same case number. Additionally, as evident in Fig 4-b, the non-pretrained model in Scenario 3 accurately identified 55 out of 120 frames as non-healthy (IS) and made 65 false predictions in the same case number. This comparison highlights the advanced predictive capability of both the pre-trained models in Scenario 2 and the non-pretrained model in Scenario 3 in distinguishing IS and healthy frames.

**Fig 1**. Testing results for Scenario 1 (Xception model), involving 16 LUS clips for both healthy and IS cases, with true and false predictions of LUS frames and their percentage within LUS frames. The test subset consisted of 4 cases classified as healthy (each case containing 2 LUS clips) and 4 cases with IS (each case containing 2 LUS clips).


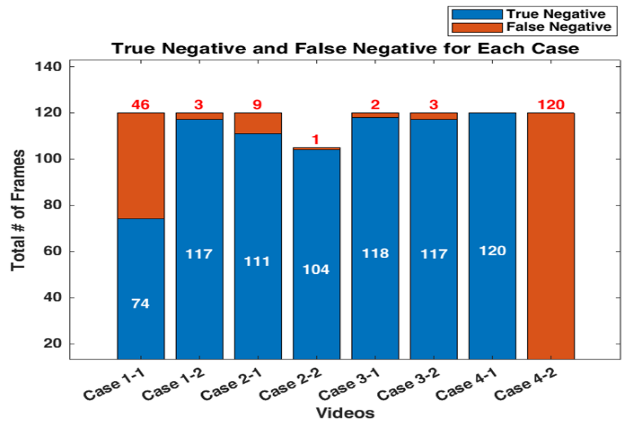

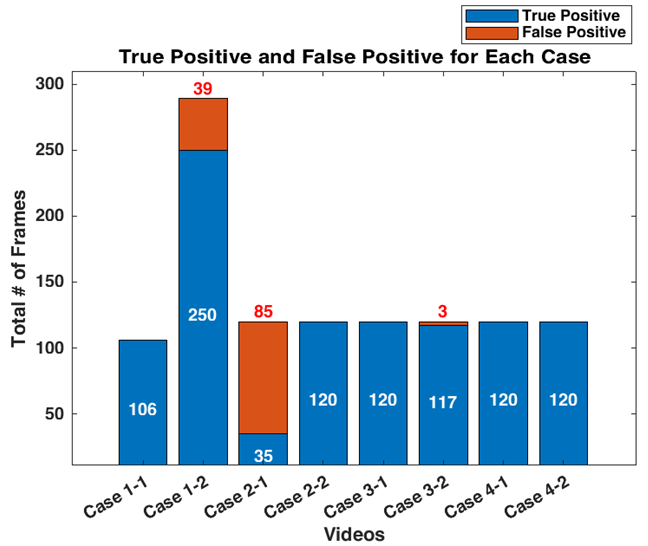


**(a)**

**(b)**

**Fig 2**. Testing results for Scenario 2 (Xception model), involving 16 LUS clips for both healthy and IS cases, with true and false predictions of LUS frames and their percentage within LUS frames. The test subset consisted of 4 cases classified as healthy (each case containing 2 LUS clips) and 4 cases with IS (each case containing 2 LUS clips).00


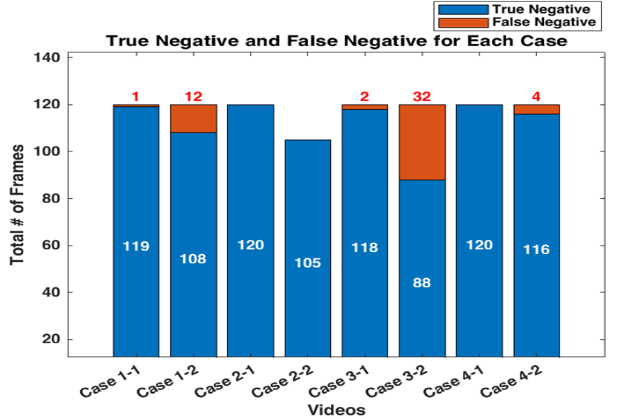

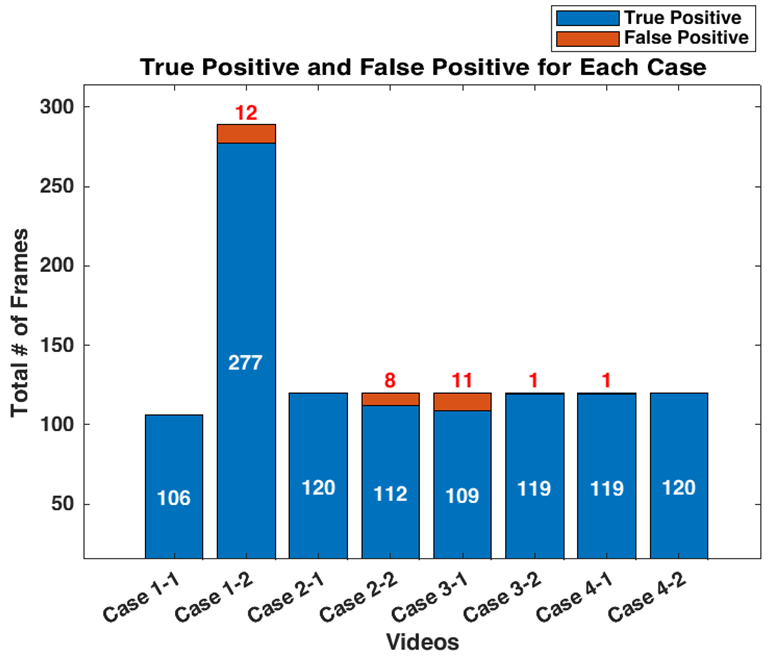


**(a)**

**(b)**

**Fig 3.** Testing results for Scenario 2 (InceptionResnetV2 model), involving 16 LUS clips for both healthy and IS cases, with true and false predictions of LUS frames and their percentage within LUS frames. The test subset consisted of 4 cases classified as healthy (each case containing 2 LUS clips) and 4 cases with IS (each case containing 2 LUS clips).


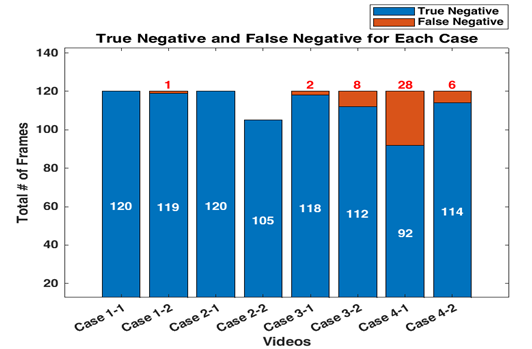

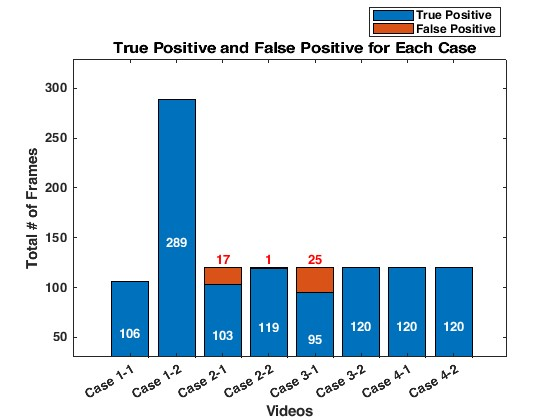


**(a)**

**(b)**

**Fig 4**. Testing results for Scenario 3 (Non-pretrained Model), involving 16 LUS clips for both healthy and IS cases, with true and false predictions of LUS frames and their percentage within LUS frames. The test subset consisted of 4 cases classified as healthy (each case containing 2 LUS clips) and 4 cases with IS (each case containing 2 LUS clips).


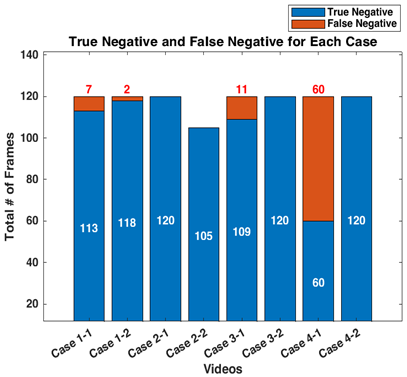

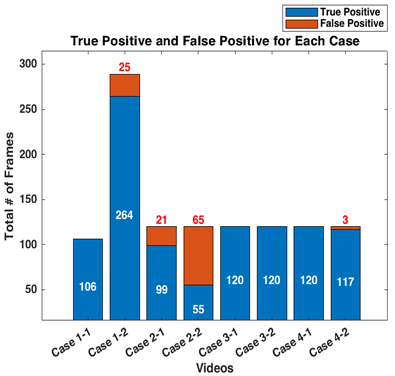


**(a)**

**(b)**

# Appendix B

**Grad-CAM (Heatmap)**

For the SIS sample in Figure 5, the heatmaps (Grad-CAM) show how the Xception model in Scenario 1 correctly detected the test sample of SIS, with a confidence value of 100%; however, focusing on areas outside the intended ROI, marked with the red box on the input image. This indicates a failure to accurately capture key ROI features. Nevertheless, both the InceptionrestnetV2 and Xception models in Scenario 2 precisely identified the sample (SIS) with a high confidence score (100%), and only the Xception model was able to detect the correct ROI properly. The non-pretrained model in Scenario 3 also precisely identified the sample as unhealthy (SIS), with a confidence value of 100%, and partially accurately detected the correct ROI. All the models correctly identified the test samples with IS according to Figure 5; however, only the Xception model in Scenario 2 focused on the ROI.

For the healthy sample in Figure 5, the heatmaps show that all models correctly predicted the test healthy LUS frames. Nonetheless, in Scenario 2, both models precisely recognised healthy samples with a greater confidence score, specifically 100%, surpassing the performance of the Xception model in Scenario 1 (78.9%) and the non-pretrained model in Scenario 3 (99.6%). In the most effective Scenario 2, the heat maps for the Xception model displayed a blue-green area (highlighted by a green box), which did not align with the ROI marked by a red box. Conversely, InceptionResnetV2's Grad-CAM accurately focused on the ROI, aligning perfectly with the area marked by the red box.

The Grad-CAM visualisation for the SIS sample in Figure 5 reveals that the Xception model in Scenario 2 accurately identifies ROI with high confidence, precisely matching the ROI. On the other hand, the Grad-CAM visualisation for the healthy sample highlights the InceptionResnetV2 model's capabilities in Scenario 2, where it not only predicts with accuracy and high confidence but also focuses on the ROI, mirroring the area observed in the ROI in the input images.

**LIME (Interpretability)**

Figure 6 presents the LIME comparison of all models in the scenarios, using two examples: one true positive (SIS) and one true negative (Healthy). For the true positive, the figure illustrates their accurate identification of the test frames as correct predictions by all models. However, in the case of the Xception model Scenario 1, the figure displays that the high-intensity area is located within the ROI. Although Scenario 1 correctly predicted the class with a reasonable confidence value (87.5%), the LIME visualisation clearly shows that the region with the highest intensity matches the ROI; however, the LIME visualisation shows a more diffuse pattern. In contrast, Scenario 2's models demonstrated a significant confidence improvement with a targeted and refined focus of LIME on the ROI. The models therein confidently predict the input frame with a confidence score of 100%. The accompanying LIME visualisation further confirms the Xception model's confidence by accurately identifying the ROI, as evidenced by the assignment of the maximum intensity value to the appropriate area. However, in InceptionResnetV2 in Scenario 2, the LIME visualisation shows the strongest signal at the top area of the image. This area is outside the expected ROI, suggesting that the model attributes high importance to features not diagnostically relevant for IS. The accuracy of Xception and InceptionrestnetV2 models in Scenario 2 is significantly enhanced, as indicated by the confidence score of 100%. The LIME visualisation for the Xception model aligns perfectly with the ROI, indicating a high degree of accuracy.

**Figure 5**. Visualisation of Grad-CAM and confidence values for the LUS frame predicted for SIS and Healthy frames for all models. The red box in the input image indicates the intended Region of Interest (ROI). In each scenario, the images display the Grad-CAM with a red box highlighting the ROI. Each image displays the Grad-CAM with a red box indicating the intended ROI. A red cross is shown if the Grad-CAM does not align with the ROI, and a green checkmark is shown if it does.


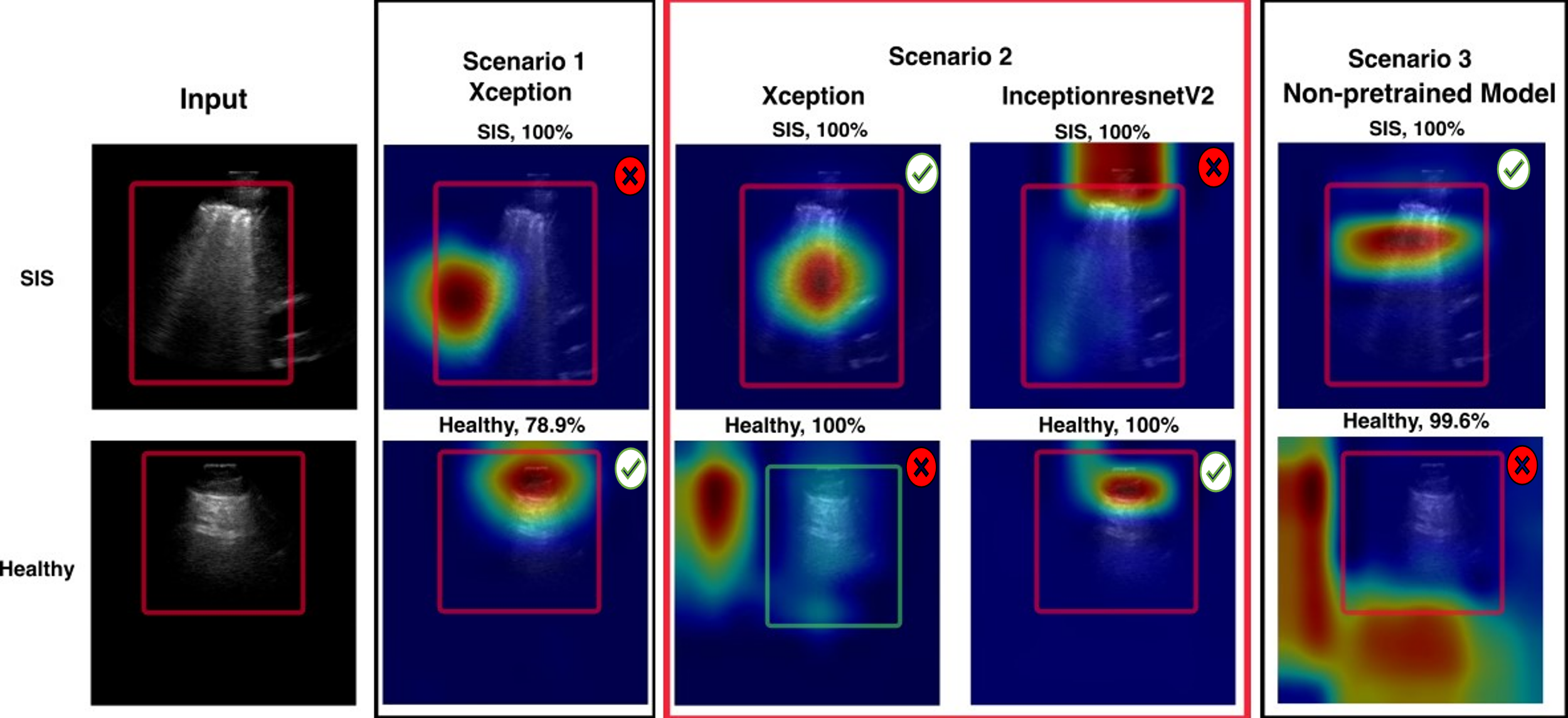


**Figure 6**. Visualisation of LIME and confidence values for LUS frame predicted as SIS and Healthy frames for all models. For SIS sample, Scenario 1 model focuses on multiple areas, shown as diffuse pattern, marked with green, red, and blue colours, while Scenario 2 models’ focuses on IS features (yellow arrows) within the intended ROI (marked with the red box on the input image). For the healthy sample, Scenario 1 model focuses on multiple areas, marked in in blue and red, while Scenario 2 focuses on IS features (yellow arrows) within the intended ROI, marked with red and dark green.


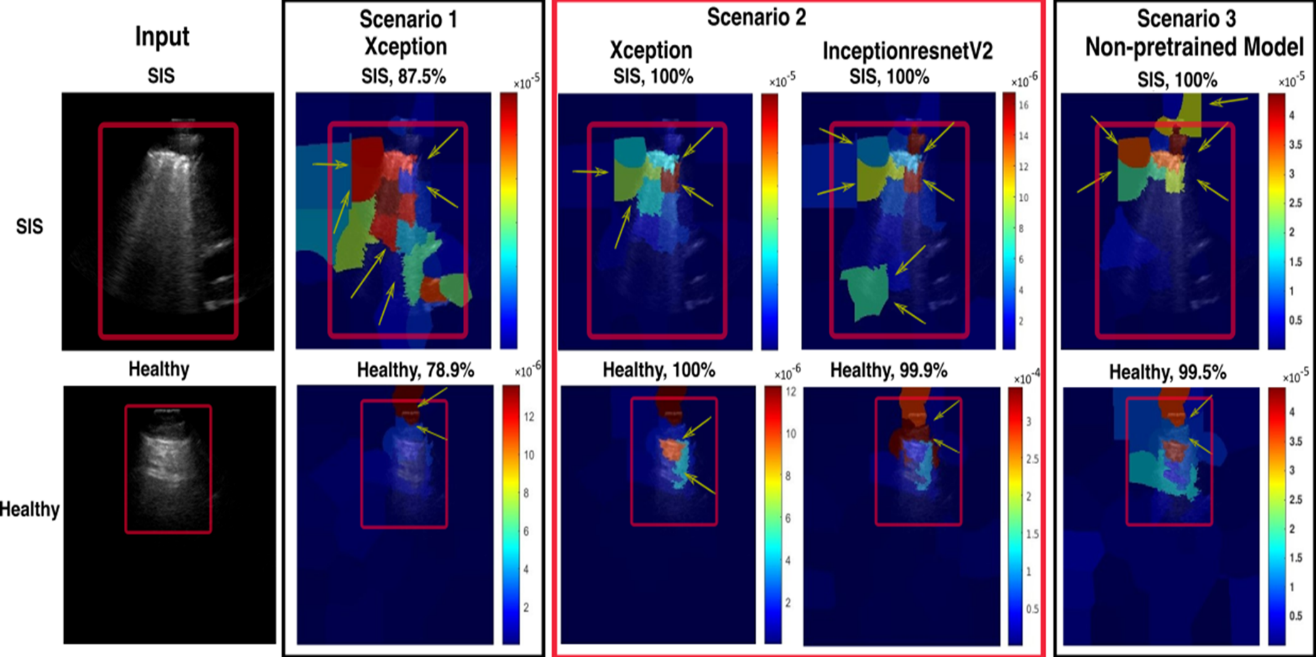


In contrast, and notably for the InceptionResnetV2 model, it indicates the higher intensity signals in the heatmap, primarily focused on the ROI and extended to areas outside of it. Furthermore, the non-pretrained model in Scenario 3 also confidently predicts the input frame with a confidence score of 100%. However, the LIME visualisation shows the strongest signal at the top area of the image, as shown in Figure 6.

For the true negative, in Figure 6, the LIME visualisation shows that all models exhibit medium to high confidence values, where Scenario 1's model achieved confidence values of 78.9%. In comparison, the Xception model in Scenario 2 achieved a confidence value of 100%. The InceptionrestnetV2 model in Scenario 2 achieved confidence values of 99.9%, whereas the non-pretrained model in Scenario 3 achieved confidence values of 99.5%. Despite their closely matched overall confidence values, the models differ in localising ROI with varying intensity values. Scenario 1's model prioritised features representing healthy attributes with low-intensity values (depicted in blue and red). On the contrary, Scenario 2's models effectively captured crucial 'healthy' features characterised by high-intensity values (shown in red and dark green). Notably, Scenario 2's models successfully highlighted these essential healthy features, ensuring a precise alignment of high-intensity areas with the ROI.

|  | H | *IS* |  | | | |
| --- | --- | --- | --- | --- | --- | --- |
| **Frame no.** | ***189*** | **223** |  |  |  |  |
| **Total frame no** | **412** | | **H** | **IS** | **H** | **IS** |
|  | *Correct class*/ accuracy | | Correct ROI: Grad-Cam/ accuracy | | Correct ROI: LIME/accuracy | |
| Xception Scenario 1 | 151/*189 (79.89%)* | 198/223 (88.79%) | 126/151 (83.44%) | 146/198 (73.74%) | 151/151 (100%) | 113/198 (57.07%) |
| Total correct | *349/*412 (84.71%) | | 272/394 (69.04%) | | 264/394 (67.01%) | |
| Xception Scenario 2 | **178*/189 (94.18%)*** | **215/223 (96.41%)** | 0/178 (0.0%) | 215/215 (100%) | **175/178 (98.31%** | **215/215 (100%)** |
| Total correct | **393/412 (95.39%)** | | 215/393 (54.71%) | | **390/393 (99.24%)** | |
| InceptionResnetV2 Scenario 2 | **180*/189 (95.24%)*** | **214/223 (95.96%)** | 166/180 (92.22%) | 0/214 (0.0%) | **180/180 (100%)** | **214/214 (100%)** |
| Total correct | **394/412 (95.96%)** | | 166/394 (42.13%) | | **394/394 (100%)** | |
| Non-pretrained model Scenario 3 | 169*/189 (89.42%)* | 200/223 (89.69%) | 101/169 (59.76%) | 200/200 (100%) | **166/169 98.22%** | **200/200 (100%)** |
| Total correct | 369/412 (89.56%) | | 301/369 (81.57%) | | **366/369 (99.19%)** | |

**Table 1:** Performance of models in Scenario 1, Scenario 2, Scenario 3 based on three measures: Overall Accuracy, Grad-CAM Accuracy, and LIME Accuracy. The table presents the number of accurate predictions and the accuracy of ROI localizations made by Grad-CAM and LIME, along with the corresponding accuracy percentages

# Appendix C

Figures 7, 8, 9, and 10 show a comprehensive overview of the ML classifiers' performance on the test set, 16 LUS clips, with a total frame number of 2060 (1115 IS frames and 945 healthy). The figures below show the model classification performance for each clip, including the count of LUS frames and true and false predictions for healthy and non-healthy frames. For example, in case number 2-2 from Figure 7-b, which is labelled as IS, the first classifier (C1) accurately identified 109 out of 120 frames as non-healthy (IS) while incorrectly predicting 11 frames as healthy. In contrast, considering the same case number, the second classifier (C2), as indicated in Figure 8-b, performed better by correctly predicting all frames as non-healthy (IS). Furthermore, according to Figure 9-b, the third classifier (C3) correctly frames as non-healthy (IS) and made 16 incorrect predictions in the same case number. Additionally, as evident in Figure 10-b, the fourth classifier (C4) only accurately identified 99 out of 120 non-healthy (IS) frames and made 21 false predictions in the same case number. This comparison highlights the advanced predictive capability of fused pre-trained models in the second fusion process (F2), which incorporates features from both Xception and InceptionResnetV2 in Scenario 2 for accurately differentiating between IS and healthy frames.

**Figure 7**. Testing results for the first classifier (C1), involving 16 LUS clips for both healthy and IS cases, with true and false predictions of LUS frames and their percentage within LUS frames. The test subset consisted of 4 cases classified as healthy (each case containing 2 LUS clips) and 4 cases with IS (each case containing 2 LUS clips).


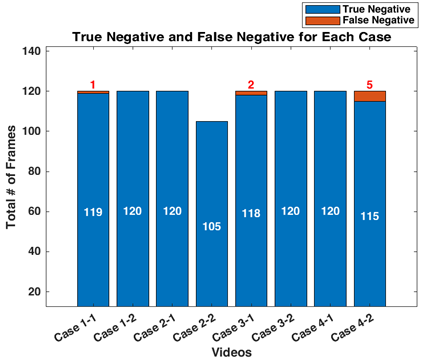

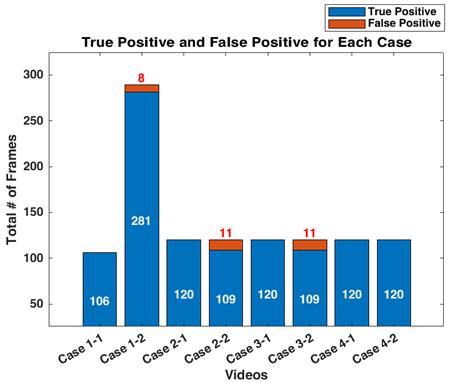


**(a)**

**(b)**

**Figure 8**. Testing results for the second classifier (C2), involving 16 LUS clips for both healthy and IS cases, with true and false predictions of LUS frames and their percentage within LUS frames. The test subset consisted of 4 cases classified as healthy (each case containing 2 LUS clips) and 4 cases with IS (each case containing 2 LUS clips).


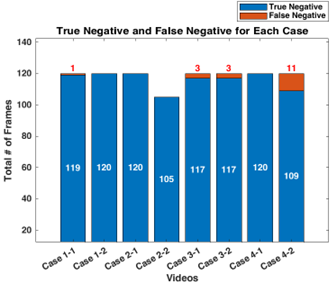

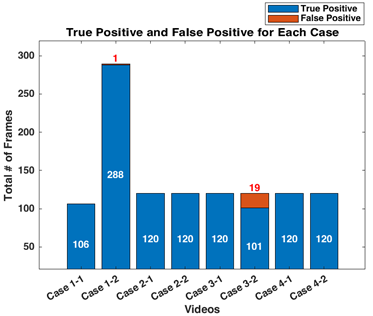


**(a)**

**(b)**

**Figure 9**. Testing results for the third classifier (C3), involving 16 LUS clips for both healthy and IS cases, with true and false predictions of LUS frames and their percentage within LUS frames. The test subset consisted of 4 cases classified as healthy (each case containing 2 LUS clips) and 4 cases with IS (each case containing 2 LUS clips).


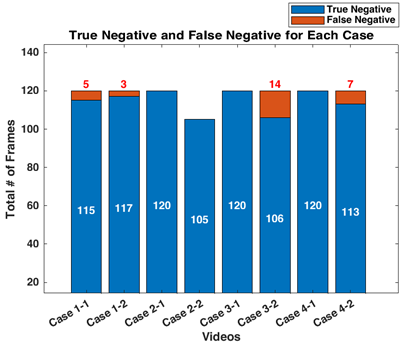

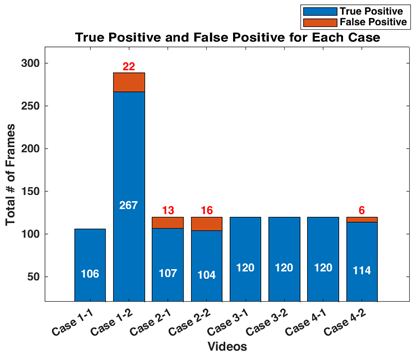


**(a)**

**(b)**

**Figure 10**. Testing results for the fourth classifier (C4), involving 16 LUS clips for both healthy and IS cases, with true and false predictions of LUS frames and their percentage within LUS frames. The test subset consisted of 4 cases classified as healthy (each case containing 2 LUS clips) and 4 cases with IS (each case containing 2 LUS clips).


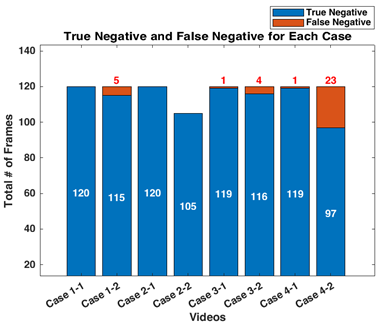

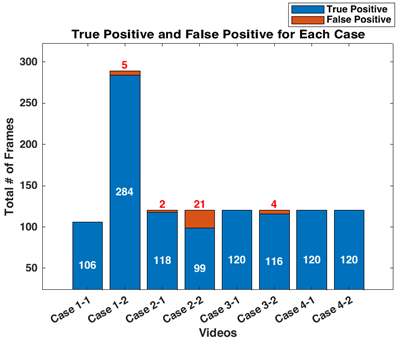


**(a)**

**(b)**

# Appendix D

Figure 11 shows examples of IS frames extracted from a sample LUS clip. The model in Scenario 1 had confidence values (85%, 92.7%, 99.5%, and 98.8%) in four frames, which correctly predicted IS examples. Both models in Scenario 2 showed high confidence scores; however, two frames were mispredicted as healthy. On the other hand, confidence values with all the same frames were predicted correctly (100%). Furthermore, the non-pretrained model in Scenario 3 correctly predicted the same frames as IS, with confidence values ranging from 77.3% to 100%.

**Figure 11.** A comparison of confidence values in Scenarios 1, 2, and 3. The models in Scenario 2 and Scenario 3 performed better than the model in Scenario 1, which misclassified two frames as healthy. Both models in Scenario 2 achieved the highest accuracy, correctly predicting all frames with high confidence (100%). The model in Scenario 3 also correctly predicted all frames but with lower confidence values (77.3%–100%). The two red rectangles illustrate the mislabelled frames by the model in Scenario 1.


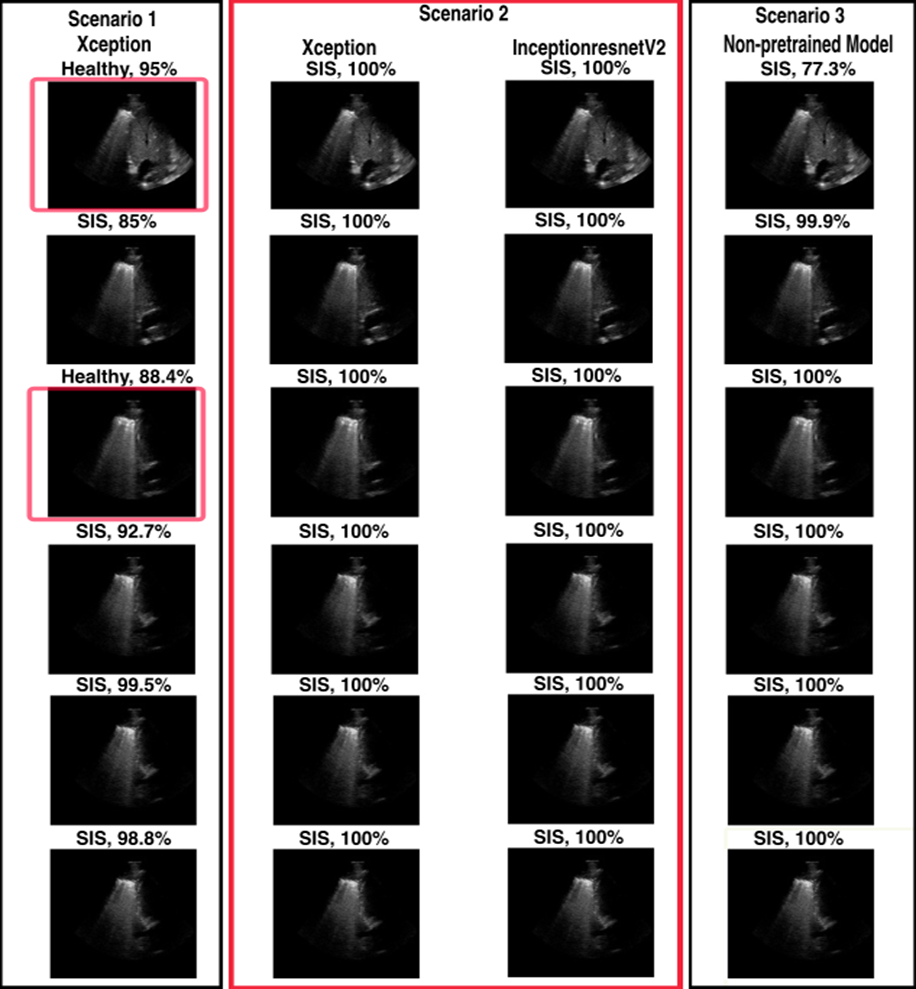

Supplement: Supplementary file 1 [file Supplementaryfile1.docx]
